# *Salmonella* Genomic Island 1 requires a self‐encoded small RNA for mobilization

**DOI:** 10.1111/mmi.14846

**Published:** 2021-11-25

**Authors:** István Nagy, Mónika Szabó, Anna Hegyi, János Kiss

**Affiliations:** ^1^ Institute of Genetics and Biotechnology Hungarian University of Agriculture and Life Sciences Gödöllő Hungary; ^2^ Present address: Heim Pál National Pediatric Institute Budapest Hungary

**Keywords:** conjugation, horizontal gene transfer, IncC plasmids, multiresistance, SGI1, small RNA

## Abstract

The SGI1‐family elements that are specifically mobilized by the IncA‐ and IncC‐family plasmids are important vehicles of antibiotic resistance among enteric bacteria. Although SGI1 exploits many plasmid‐derived conjugation and regulatory functions, the basic mobilization module of the island is unrelated to that of IncC plasmids. This module contains the *oriT* and encodes the mobilization proteins MpsA and MpsB, which belong to the tyrosine recombinases and not to relaxases. Here we report an additional, essential transfer factor of SGI1. This is a small RNA deriving from the 3′‐end of a primary RNA that can also serve as mRNA of ORF S022. The functional domain of this sRNA named sgm‐sRNA is encoded between the *mpsA* gene and the *oriT* of SGI1. Terminator‐like sequence near the promoter of the primary transcript possibly has a regulatory function in controlling the amount of full‐length primary RNA, which is converted to the active sgm‐sRNA through consecutive maturation steps influenced by the 5′‐end of the primary RNA. The mobilization module of SGI1 seems unique due to its atypical relaxase and the newly identified sgm‐sRNA, which is required for the horizontal transfer of the island but appears to act differently from classical regulatory sRNAs.

## Graphical abstract

The SGI1‐family mobilizable genomic islands and the IncA‐ and IncC‐family plasmids that specifically mobilize the SGI1‐like elements are major distributors of antibiotic resistance among enteric bacteria. The sophisticated interactions between SGI1 and its helper plasmid guarantee the efficient horizontal transfer and stability of SGI1 in the host cell. Interestingly, the basic mobilization module of SGI1, including *oriT* and two mobilization proteins, is unrelated to that of IncC plasmids. Here we report an additional, essential transfer factor encoded by SGI1, which is a small RNA. The mobilization module of SGI1 appears unique due to its atypical relaxase and the newly identified sRNA involved in the horizontal transfer of the island.

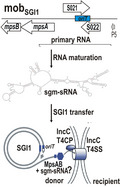

## 1 INTRODUCTION

The worldwide spread of multidrug‐resistant (MDR) bacteria is one of the biggest threats to public and livestock health as well as to food security (WHO). The most efficient distributors of resistance determinants are the conjugative plasmids and the integrative elements (IE), for example, the autonomous conjugative (ICE) and the nonautonomous mobilizable (IME) elements, which need a conjugative helper element (plasmid or ICE) for their horizontal transfer (Bellanger et al., 2014).

Conjugation, which requires close cell‐to‐cell contact, is a universal mechanism for horizontal gene transfer among bacteria. The process in Gram‐negatives begins with the assembly of a multi‐protein–DNA complex called relaxosome on the *cis*‐acting DNA region, the origin of transfer (*oriT*), followed by nicking either strand of the plasmid or IE DNA at the *nic* site of *oriT* by the relaxase. Subsequently, the relaxase and the covalently bound single‐stranded DNA are delivered to the type IV secretion system (T4SS) with the assistance of the membrane‐associated coupling protein (T4CP), which binds the cognate T4SS and the relaxosome complex. Finally, the transported DNA strand is recircularized and converted to the double‐strand form in the recipient cell (Bellanger et al., 2014; Llosa & Alkorta, 2017; Llosa et al., 2002). Relaxases, the key enzymes of conjugative transfer are currently classified into eight families (Guzmán‐Herrador & Llosa, 2019). Four of them (MOB_F_, MOB_Q_, MOB_P_, and MOB_V_) include HUH nucleases (Chandler et al., 2013) having a conserved motif of three amino acid residues (His, U = a hydrophobic residue, His) responsible for the binding of divalent cations. MOB_C_ proteins are related to PD‐(D/E)XK restriction enzymes, whereas the MOB_T_ family enzymes belong to the Rep_trans nucleases (Carr et al., 2016). MOB_H_ relaxases show some sequence similarities to HUH proteins but are related to HD‐hydrolases and form a separate clade (Garcillán‐Barcia et al., 2009). Common features of most relaxases are the generation of single‐strand cleavage in *oriT* at the *nic*‐site and that they remain covalently attached to the 5′‐end. In this respect, MobC, the relaxase of plasmid CloDF13 (MOB_C_), and TraI of the *Neisseria* genomic island GGI (MOB_H_) appear to be exceptional, which probably have adopted unique molecular mechanisms for DNA cleavage without forming covalent relaxase‐DNA intermediates (Heilers et al., 2019; Núñez & De La Cruz, 2001). The newest group of relaxases includes enzymes related to the Tyr‐recombinases. Currently, only two plasmid‐derived proteins, TcpM and MobK, the relaxases of pCW3 and pIGRK, respectively, and MpsA protein, the presumed relaxase of the *Salmonella* IME, SGI1, belong to this group (Kiss et al., 2019; Nowak et al., 2021; Wisniewski et al., 2016).

**FIGURE 1:**
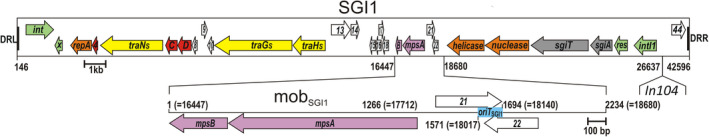
Schematic maps of SGI1 backbone and the mob_SGI1_ region. The annotated ORFs originally designated as S001–S044 are indicated by arrows (the color coding is used accordingly throughout the figures): green, recombinase; orange, replication, DNA processing; red, regulator; yellow, T4SS components; gray, TA system; purple, SGI1 mobilization; white, unknown function (these ORFs are numbered according to their original numbering, e.g., “8” refers to S008). Abbreviations: *x*—*xis*, *C* and *D*—*flhC*_SGI1_ and *flhD*_SGI1_, *B*—*mpsB*. Terminal direct repeats DRL and DRR are shown as black boxes. The insertion site of In104 is indicated. Coordinates refer to the published SGI1 sequence AF261825. The zoomed map of the mob_SGI1_ region including *oriT*_SGI1_ (blue box), and four ORFs is shown below the full SGI1 map. The coordinates of mob_SGI1_ (used throughout the figures) are numbered from the last base of the STOP codon of *mpsB*: the first bp of mob_SGI1_ corresponds to the 16447th bp of AF261825 sequence). START of *mpsA* and the termini of *oriT*_SGI1_ are also indicated. Maps are drawn to scale in all figures

IMEs have recently been recognized as key players in the distribution of resistance determinants to antibiotics and heavy metals, virulence factors, or even gene sets for metabolic pathways or transport systems (Bellanger et al., 2014). The *Salmonella* Genomic Island 1 (SGI1), its variants, and related elements that form a large family of IMEs are often responsible for the MDR phenotype of human pathogens like *Salmonella enterica* serovars, *Proteus mirabilis*, *Morganella morganii*, *Acinetobacter baumannii*, *Providencia stuartii, Enterobacter* spp., *Escherichia coli*, or *Klebsiella pneumoniae* strains (Cummins et al., 2019, 2020; Schultz et al., 2017; Siebor et al., 2016, 2019; Soliman et al., 2018, 2020). SGI1‐family elements integrate at the 3′‐end of the chromosomal GTPase gene *trmE* (also known as *thdF* or *mnmE*) and share a conserved backbone (Figure 1) including genes for integration/excision (*int* and *xis*), the replication module (*repA*, S004, and *oriV*) (Szabó et al., 2021), T4SS subunits (*traN_S_*, *traG_S_*, and *traH_S_*) (Boyd et al., 2001), a pair of genes encoding FlhDC‐family activators (Kiss et al., 2015), a mobilization module (*mpsA*, *mpsB*, and *oriT*) (Kiss et al., 2019), a TA system (Huguet et al., 2016), genes for a helicase, a nuclease, and a resolvase (*res*) and several ORFs with unknown functions (S008–S010, S013–S018, S021–S022, and S044) (Boyd et al., 2001). Most SGI1 variants carry a complex In4‐type integron structure (In104) containing diverse sets of antibiotic resistance (AR) genes. In104 is generally inserted into the SGI1 backbone between the *res* and S044 genes, however, SGI2 carries the integron cluster within its helicase gene (S023) (Levings et al., 2008) and several relatives lack integrons at all (Cummins et al., 2020; de Curraize et al., 2020).

SGI1‐family elements are mobilized in trans by the large single‐copy conjugative plasmids of the closely related IncA and IncC groups (Ambrose et al., 2018; Douard et al., 2010; Harmer & Hall, 2015). These broad host range plasmids are prevalent in Gram‐negatives and seem to be key players in the distribution of many AR genes including novel metallo‐β‐lactamase genes among clinical isolates of *Enterobacteriaceae* (Wu et al., 2019; Zhang et al., 2020; Zheng et al., 2020). The conjugative system of IncA and IncC plasmids is classified into the MOB_H12_ group (Garcillán‐Barcia et al., 2009) and controlled by the FlhDC‐family master activator, AcaCD, which activates 18 promoters in the IncC backbone, including promoters of the entire conjugative apparatus and several genes of unknown functions (Carraro et al., 2014; Durand et al., 2021b). SGI1 exploits this conjugative system and its control mechanisms in multiple ways. AcaCD activates five operons of SGI1 including *xis*, *rep*, *traN_S_*, and *traG_S_H_S_*, which ensure the efficient horizontal transfer and stability of the island (Carraro et al., 2014, 2017; Huguet et al., 2020; Kiss et al., 2015; Szabó et al., 2021). Furthermore, SGI1 also encodes an FlhDC‐family activator, FlhDC_SGI1_ (also known as SgaDC) (Kiss et al., 2015), which acts on the AcaCD‐responsive promoters of SGI1 and the IncC plasmids (Murányi et al., 2016). FlhDC_SGI1_ encroaches on the regulatory circuits of IncC transfer and appears to have a key role in the parasitism by SGI1 on IncC plasmids (Durand et al., 2021a, 2021b; Kiss et al., 2015; Szabó et al., 2021).

Although SGI1 encodes three T4SS subunits (TraN_S_, TraG_S_, and TraH_S_), they are not essential for SGI1 transfer (Kiss et al., 2012). Instead, their role is apparently to ensure advantages for SGI1 over the helper plasmid during the transfer (Carraro et al., 2017). On the other hand, SGI1 has its own mobilization unit (mob_SGI1_, Figure 1) that is unrelated to the transfer apparatus of the helper plasmids or to any other known conjugation systems. The mob_SGI1_ region contains the *mpsAB* operon coding for two proteins that are indispensable for SGI1 transfer, and the *oriT*_SGI1_ not resembling that of IncC plasmids (Hegyi et al., 2017; Kiss et al., 2019). Furthermore, SGI1 transfer does not absolutely depend on the IncC relaxase, as it can occur even in the lack of TraI of the helper, although, with lower frequency. MpsA protein belongs to the Tyr‐recombinase/integrase superfamily and is unrelated to other relaxase families. The conserved catalytic tyrosine residue characteristic for Tyr‐recombinases was predicted at the C‐terminus of MpsA (Y319) and R162, H247, R250, H251 matching with the catalytic residues of well‐characterized recombinases, such as Hp1 Int, Cre, λ Int, and IntI4, were also identified. MpsB contains a phage integrase N‐terminal SAM‐4‐like domain resembling the N‐terminal core‐binding domain of λ integrases. MpsA and two plasmid‐borne relaxases, TcpM and MobK, appear to be Tyr‐recombinase‐like proteins, which is mostly based on the similarity of their catalytic domains to the DNA_BRE_C domains with the conserved catalytic pentad RK(H/Y)YRH. However, they are not related to each other and the genetic context of the respective mob regions is also different, except that the *oriT*s are located upstream of the relaxase genes (Kiss et al., 2019; Nowak et al., 2021; Wisniewski et al., 2016). The three proteins lack the core DNA‐binding domain that is obligatory to the classical Tyr‐recombinases suggesting that they bind to their cognate *oriT*s differently. In the case of SGI1, MpsB may be an accessory protein involved in DNA binding as it resembles the core‐binding domains of integrases (Kiss et al., 2019; Nowak et al., 2021); however, similar accessory proteins have not yet been identified on pCW3 and pIGRK.

Even though the main factors required for SGI1 transfer (e.g., the SGI1‐encoded proteins and the *cis*‐acting element *oriT*_SGI1_) have been identified (Kiss et al., 2019), the molecular mechanisms of transfer initiation and the components of the relaxosome are still unknown. In the present study, we report a new, essential transfer factor expressed by SGI1. Based on phenotype analyses of deletion mutants, transcomplementation assays, Northern analyses, and different methods for determining transcription start sites (TSS), we show that the transfer factor is a small RNA (sRNA) expressed from a promoter region located upstream of ORF S022. The functional part of this sRNA has been determined and its possible function is discussed.

## 2 RESULTS

### 2.1 Discovery of a new factor required for mobilization of SGI1

**FIGURE 2:**
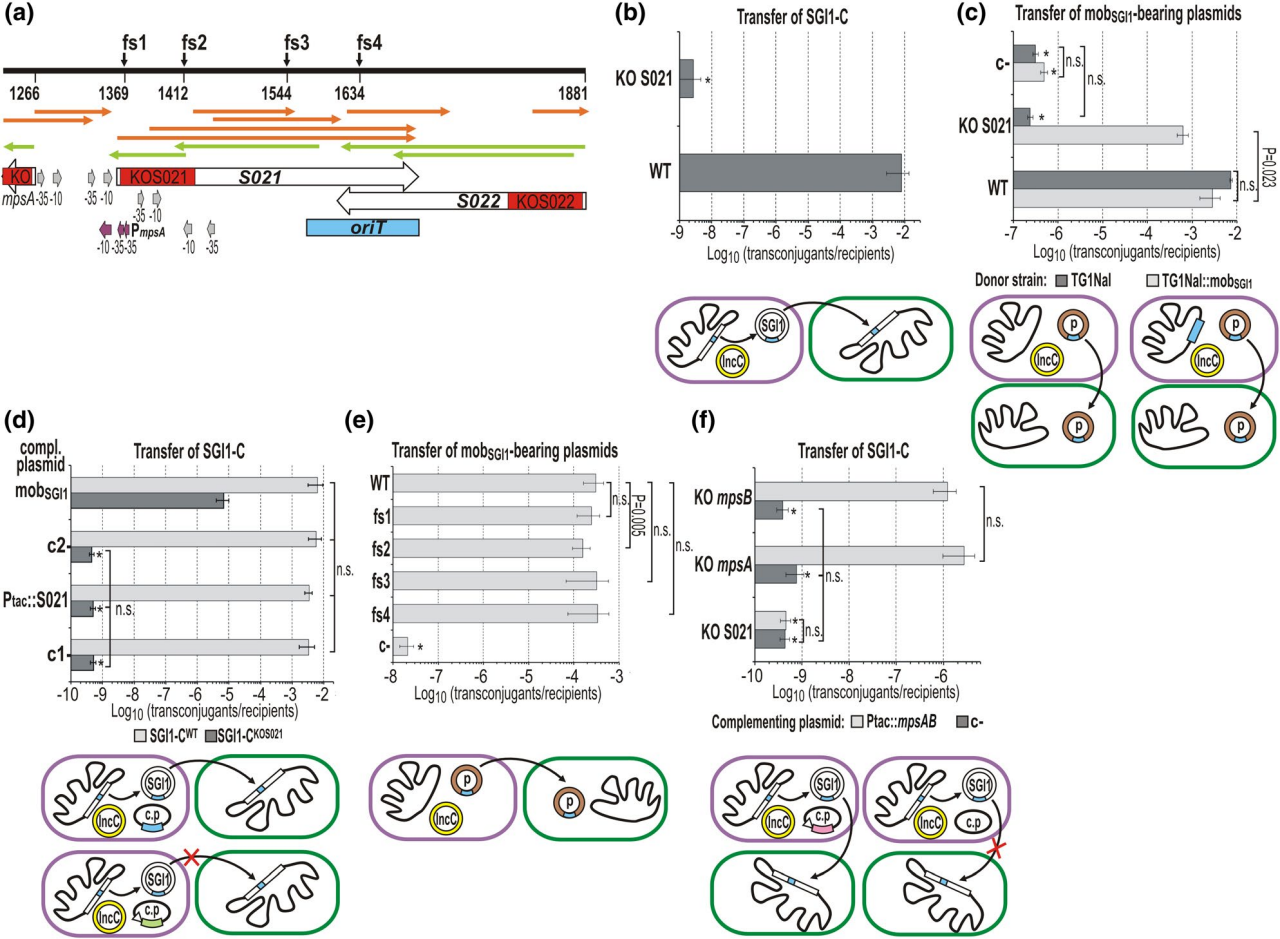
Mobilization and complementation of S021 KO mutants. (a) Schematic map of the S021‐*oriT*‐S022 region of mob_SGI1_. The positions of the replacement KO mutations are indicated in the ORFs by red boxes (*mpsA* gene and its KO mutation are shown partially in the map). Frameshift mutations **fs1–fs4** were introduced to the examined region and their exact positions are indicated (coordinates are as defined for mob_SGI1_ region in Figure 1). Gray arrows show the putative promoter boxes located near the KOS021 mutation. The possible promoter boxes of *mpsA* are shown as purple arrows. ORFs longer than 25 codons are indicated by orange and light green arrows. The mating assays shown in Panels (b)–(f) were carried out using different derivatives of the *E. coli* strain TG1Nal (Nal^R^) as donors and TG2 (Tc^R^) as the recipient, if not otherwise specified. All donor strains harbored either R55 (Km^R^Gm^R^Cm^R^Flo^R^Ap^R^Sul^R^) or R55^ΔTn^ *^6187^* (Cm^R^Flo^R^Sul^R^) as a mobilization helper. The donors in the different assays carried also wt/mutant SGI1‐C (Sm^R^Sp^R^Sul^R^) or wt/mutant mob_SGI1_‐bearing test plasmids (Sm^R^Sp^R^ or Km^R^) or wt/mutant SGI1‐C along with one of the complementing plasmids (Km^R^). The pictograms below the graphs explain the particular experimental setup. Only the measured transfer events are indicated by arrows (e.g., conjugation of the helper plasmid or the mob_SGI1_‐bearing plasmid when used as complementing plasmid are not shown). Symbols used accordingly throughout the figures are as follows: purple oval—donor cell, green—oval recipient cell, black curve—chromosome, open box—chromosomally integrated SGI1‐C, open circle—excised SGI1‐C, yellow circle—IncC helper plasmid, blue box—mob_SGI1_, brown circle—test plasmid (p), black oval—complementing plasmid (c.p.), open arrowhead—P_tac_ promoter, ORFs expressed for complementation: light green box—S021, purple box—*mpsAB*. Transfer frequencies are expressed as transconjugant per recipient CFUs. For transconjugant/donor frequency data, see Figure S1. Paired *t* test was used in all matings to calculate the significance of the differences, n.s.—not significant (*p* ≥ .05). Asterisks, unless otherwise specified, indicate that the transconjugant frequency was below the detection limit, transconjugants were not observed. (b) Transfer frequency of SGI1‐C^KOS021^ mutant compared with the SGI1‐C^WT^. The donor strains were TG1Nal/R55 containing wt or KO S021 SGI1‐C (Sm^R^Sp^R^Sul^R^). The SGI1‐C transconjugants were selected on TcSp plates. (c) Transfer of test plasmids carrying wt or S021 KO mutant mob_SGI1_ region. The donor strains containing R55 and one of the test plasmids were TG1Nal or the complementing strain TG1Nal::mob_SGI1_ (Nal^R^Km^R^) harboring chromosomally integrated wt mob_SGI1_ region. The wt and KO mutant mob_SGI1_ regions were introduced in the test plasmids pFOL1372 and pJKI773, respectively, whereas the empty plasmid vector pJKI708 was used as a negative control (c‐). The resistance marker of the test plasmids was Sm^R^Sp^R^. The transconjugants were selected on TcSp plates. Asterisks indicate the basal level of plasmid transfer (the empty plasmid vector pJKI708 was also mobilized at a very low frequency possibly through a sequence mimicking *oriT* of SGI1 or R55). (d) Transcomplementation of SGI1‐C^KOS021^ by the expressed S021 protein. The expression plasmid pJKI881 containing the P_tac_::S021 cassette provided the S021 protein, the positive control pMSZ949 contained the entire mob_SGI1_ region. The respective empty vectors pJKI391 and pJKI88 were used as negative controls (c1 and c2). The expression vectors pJKI881 and pJKI391 were induced with 0.05 mM IPTG. The donor strains were TG1Nal::SGI1‐C^KOS021^/R55^ΔTn^ *^6187^* (Nal^R^Sm^R^Sp^R^Sul^R^Cm^R^Flo^R^) harboring one of the complementing plasmids (Km^R^). The SGI1‐C^KOS021^ transconjugants were selected on TcSp plates. (e) Transfer frequencies of the frameshift mutant mob_SGI1_‐bearing test plasmids. The TG1Nal/R55^ΔTn^ *^6187^* (Nal^R^Sul^R^Cm^R^Flo^R^) donor strains harbored one of the Km^R^ test plasmids (wt—pMSZ949, **fs1**—pMSZ957, **fs2**—pMSZ958, **fs3**—pMSZ967, **fs4**—pAHG36, c‐—pJKI88). *E. coli* TG90 (Tc^R^) was used as a recipient. The transconjugants were selected on TcKm plates. (f) Transcomplementation of SGI1‐C^KOS021^ by co‐expression of MpsA and MpsB proteins. The complementing plasmid pJKI882 carried the P_tac_::*mpsAB* cassette, whereas the empty vector pJKI391 was the nonexpressing control (c‐). MpsAB expression was ensured by leaking P_tac_ promoter without IPTG induction. To show that pJKI882 expresses fully active MpsAB proteins under these conditions, SGI1‐C^KO^ *^mpsA^* and SGI1‐C^KO^ *^mpsB^* mutants were also complemented. The donor strain TG1Nal/R55^ΔTn^ *^6187^* (Nal^R^Sul^R^Cm^R^Flo^R^) contained one of the SGI1‐C KO mutants (Sm^R^Sp^R^Sul^R^) and the complementing plasmid pJKI882 (Km^R^). The SGI1‐C^KO^ transconjugants were selected on TcSp plates

Our previous analysis proved that the 2.2 kb mob_SGI1_ region carries all self‐encoded *cis‐* and *trans*‐acting elements for mobilization of SGI1 by the IncC plasmids (Figure 1). This region contains four annotated ORFs (*mpsA*/S020, *mpsB*/S019, S021, and S022) and the *oriT*_SGI1_, which is localized within the overlapping 3′ parts of S021 and S022 (Kiss et al., 2019). As a first step during the functional analysis of mob_SGI1_, KO mutations were generated in all four ORFs in a chromosomally integrated SGI1‐C by the one‐step gene inactivation method (Datsenko & Wanner, 2000). The KO mutagenesis resulted in the replacement of 83‐bp near the 5′‐end of each ORF with 84‐bp extraneous sequence (Figure 2a) and caused a frameshift and early stop codons in the ORFs. Mobilization of the wt and KO mutant SGI1‐C by the IncC plasmid R55 was compared in a mating assay, which revealed that KO of S022 had no negative effect on SGI1 transfer, whereas KO of *mpsA*/S020 and *mpsB*/S019 was deleterious (Kiss et al., 2019). In the same experiment, S021 KO‐mutant SGI1‐C also proved to be nonmobilizable (Figure 2b). We have shown previously that plasmids containing the entire mob_SGI1_ region are mobilizable by IncC helpers and are convenient tools for analyses of different mob mutations (Kiss et al., 2019). Thus, the S021 KO mutation was also tested in this plasmid‐based system. When the S021 KO mutant mob_SGI1_‐bearing plasmid pJKI773 was mobilized by R55 from the strain TG1Nal, a similar (negative) result was obtained as with SGI1‐C^KOS021^ (Figure 2c, dark gray bars). However, when the same mating was carried out with a donor strain containing the entire mob_SGI1_ region integrated onto the chromosome, pJKI773 appeared mobilizable, although with somewhat lower frequency than the wt mob_SGI1_‐bearing control plasmid pAW1372 (Figure 2c, light gray bars). These results clearly showed that mob_SGI1_ expresses a factor that can complement the S021 KO mutation and suggested that intact ORF S021 is required for SGI1 transfer. This finding, however, was rather unexpected as it seemingly confuted our previous observation that the frameshift mutation **fs1**, which was generated by a single‐base insertion after the third codon of S021 in a mob_SGI1_‐containing plasmid and eliminated the putative S021 protein, does not influence SGI1 transfer (Kiss et al., 2019). To further examine the role of S021 protein, ORF S021 was fused to the P_tac_ promoter and a ribosome binding site (SD‐box) deriving from pKK223‐3 and the resulting expression plasmid pJKI881 was used to complement the SGI1‐C^KOS021^ in a mating assay. For positive control, the entire mob_SGI1_ region was provided on the plasmid pMSZ949. In this assay, R55^ΔTn^ *^6187^*, whose transfer functions are the same as that of the wt R55, was applied as a mobilization helper due to its reduced antibiotic resistance spectrum (Flo^R^/Cm^R^, Sul^R^), which enabled us to apply the Km^R^ complementing plasmids together with it. The results showed that SGI1‐C^KOS021^ could not be rescued by in trans expression of S021 protein, supporting the previous observations that S021 protein itself is not required for SGI1 transfer. In contrast, the entire mob_SGI1_ complemented the SGI1‐C^KOS021^ mutant, although its transfer frequency did not reach that of SGI1‐C^WT^ (Figure 2d). These data suggested that S021 KO mutation destroyed or impaired the expression of an unknown soluble factor, which is essential for SGI1 mobilization and provided by the mob_SGI1_ region.

Because the involvement of the S021‐encoded protein was excluded again, we supposed that one of the small ORFs overlapping ORF S021 (Figure 2a) is responsible for this effect. The 83‐bp segment that was replaced in S021 KO mutant may also contain putative promoter elements, which might be responsible for the expression of these ORFs (it is worth noting that none of them has an obvious SD‐box making their translation unlikely). To test this hypothesis, three further frameshift mutations were generated in mob_SGI1_ cloned in plasmid pMSZ949. The 1‐bp or 2‐bp insertions (**fs2**–**fs4**) knocked out all the short ORFs that the KO S021 mutation might affect (Figure 2a), but none of them had a notable negative effect on the plasmid mobilization, although **fs2** caused a slight decrease (Figure 2e).

Our next hypothesis was that S021 KO mutation prevents the expression of the essential mobilization proteins MpsA and MpsB. The promoter region of *mpsAB* operon was previously localized in the noncoding region between *mpsA* and S021 (Kiss et al., 2019), and the −35 box of one of the putative promoters was removed by the S021 KO mutation (Figure 2a), thus it could not be excluded that the mutation impairs the transcription of *mpsAB*. In this case, SGI1‐C^KOS021^ should be rescued by in trans expression of MpsA and MpsB. Therefore, *mpsAB* operon was placed under the control of P_tac_ promoter in a p15A‐based plasmid pJKI882, which was used for complementation of SGI1‐C^KOS021^ in a mobilization assay. Co‐expression of MpsA and MpsB from pJKI882 successfully complemented both the *mpsA* and *mpsB* KO mutant SGI1‐C but was completely inefficient in rescuing the S021 KO mutant (Figure 2f). These results suggested that the transfer factor missing in SGI1‐C^KOS021^ cannot be a protein, thus it is presumably an RNA molecule.

### 2.2 Localization of the SGI1 region required for complementation of the S021 KO mutant

**FIGURE 3:**
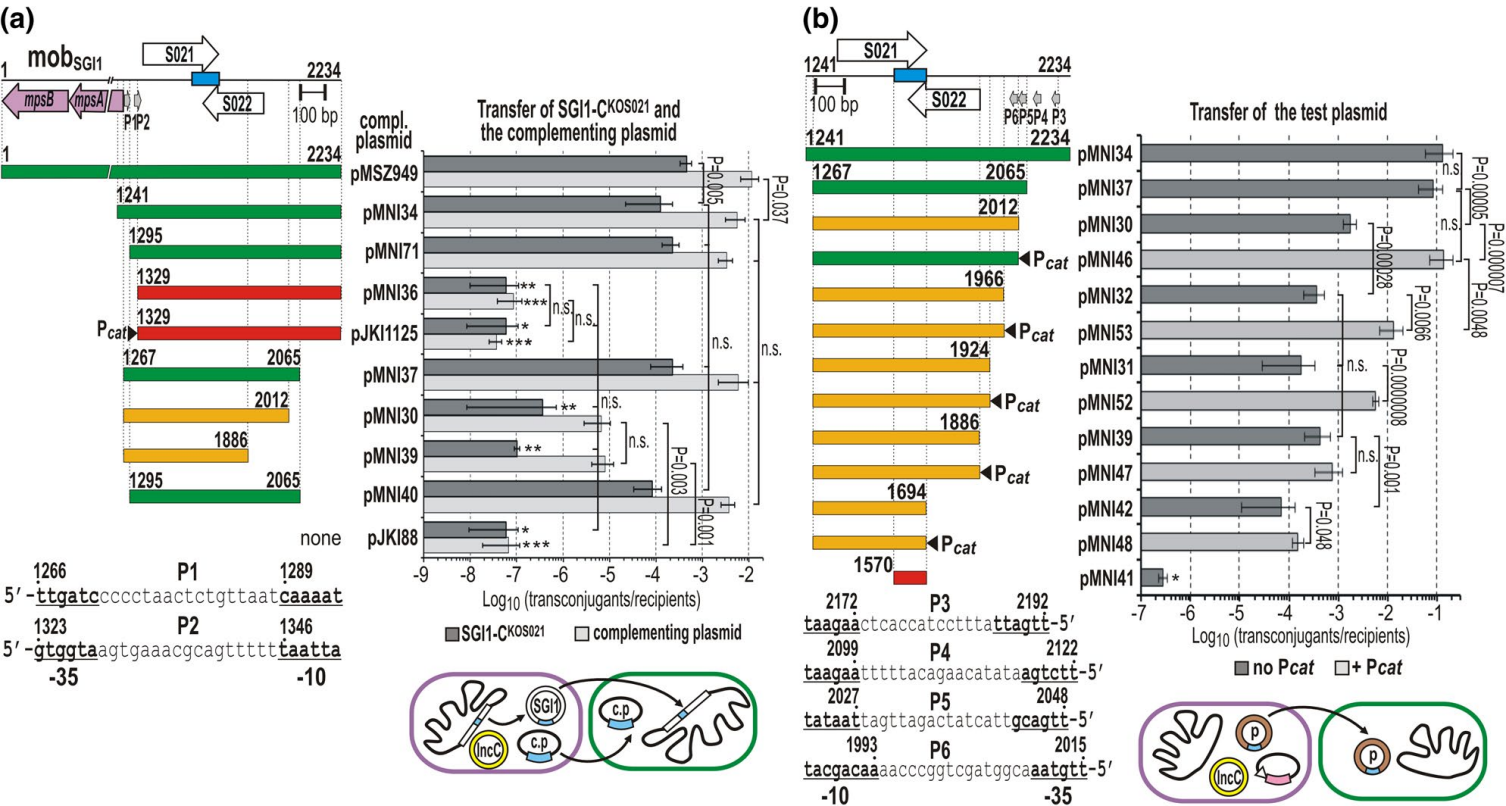
Deletion mapping of the putative RNA transfer factor in the mob_SGI1_ region. (a) Determination of the minimal SGI1 region that can complement the SGI1‐C^KOS021^ mutant. The schematic map shows the mob_SGI1_ region with the four annotated ORFs and the *oriT*_SGI1_. The predicted promoters P1 and P2 in the upstream region of ORF S021 are indicated by gray arrows and their position and sequence are shown below. The predicted −35 and −10 boxes are in bold and underlined. In the mating assay TG1Nal::SGI1‐C^KOS021^/R55^ΔTn^ *^6187^* (Nal^R^Cm^R^Flo^R^Sm^R^Sp^R^Sul^R^) transformed with one of the complementing plasmids (Km^R^) was used as donors and TG2 (Tc^R^) was the recipient. The pictogram below the graph explains the experimental setup. The transfer frequency of SGI1‐C^KOS021^ and the p15A‐based complementing plasmids was measured. Symbols are as in Figure 2. The horizontal bars below the map indicate the fragments of mob_SGI1_ cloned in the complementing plasmids, green—mobilizable and can complement SGI1‐C^KOS021^; orange—mobilizable at a reduced level and cannot complement SGI1‐C^KOS021^; red—not mobilizable. The transconjugants for SGI1‐C^KOS021^ and the complementing plasmid were selected on TcSp and TcKm plates, respectively. *The conjugation frequency was below the detection limit, no transconjugants were observed. **Several SGI1‐C^KOS021^ transconjugant colonies were obtained with a frequency around the detection limit (~10^–7^ transconjugant/recipient). ***Several plasmid transconjugants were obtained with a frequency around the detection limit (~10^–7^ transconjugant/recipient). (b) Promoter mapping and deletion analysis in the 3′‐end of *mpsAB*‐deleted mob_SGI1_ region. Gray arrows show the promoters P3–P6 predicted in the upstream region of S022. Their position and sequence are shown below the diagram. For symbols, see Panel (a) and Figure 2. *E. coli* Tuner/R55^ΔTn^ *^6187^* (Sul^R^Cm^R^Flo^R^) transformed with the MpsAB‐producer pJKI879 (Ap^R^) and one of the test plasmids (Km^R^) was used as a donor, whereas TG90 (Tc^R^) was the recipient. In the mating assay (see the pictogram below the graph), the transfer frequency of the p15A‐based test plasmids was measured. The plasmid transconjugants were selected on TcKm plates. Horizontal bars below the map indicate the mob_SGI1_ parts cloned in the test plasmids, green—mobilizable at a maximal rate; orange—mobilizable at a reduced rate; red—not mobilizable. Filled arrowheads indicate the insertion of promoter P*_cat_*. *Conjugation frequency was below the detection limit, no transconjugants were observed. For transconjugant/donor frequency data, see Figure S2

The region of mob_SGI1_ responsible for the synthesis of the hypothetical RNA and the rescue of SGI1‐C^KOS021^ was sought by complementation using a series of mob_SGI1_ subclones in a mobilization assay. These p15A‐based complementing plasmids were introduced to the donor strain harboring the R55^ΔTn^ *^6187^* helper plasmid and SGI1‐C^KOS021^ (Figure 3a). Since all the subclones contained the *oriT*, the transfer of both SGI1‐C^KOS021^ and the complementing plasmids could be monitored in the same mating assay. The maximal transfer rate and complementation of SGI1‐C^KOS021^ was observed with the entire mob_SGI1_, which was provided on pMSZ949 and used as a positive control. Removal of *mpsAB* genes from the complementing plasmid (pMNI34) had no deleterious effect as it caused only about twofold and fourfold reduction in the transfer rate of the complementing plasmid and SGI1‐C^KOS021^, respectively, compared with the positive control. This slight decrease was probably due to the partial withdrawal of MpsA and MpsB, which were now expressed only by SGI1 but utilized for the mobilization of both SGI1 and the approximately 15‐copy complementing plasmid. It is worth noting that this experiment proved again that the S021 KO mutation did not impair the expression of *mpsAB* operon as SGI1‐C^KOS021^ expressed MpsA and MpsB proteins in a sufficient amount for the transfer of both elements. Further extensive shortening of the 5′‐end of mob_SGI1_ fragment was not possible as deletion of *mpsAB* along with its 28 bp upstream sequence had no negative effect (pMNI71), but the removal of additional 34 bp (pMNI36) completely terminated the transfer of both the SGI1^KOS021^ and the complementing plasmid. On the other hand, longer DNA sequences could be deleted from the 3′‐end, which influenced the efficiency of complementation at various degrees. Removal of the last 169 bp of mob_SGI1_ had no significant effect (pMNI37), while larger deletions (222 bp and 348 bp in pMNI30 and pMNI39, respectively) abolished the mobilization of SGI1‐C^KOS021^, but the transfer of the complementing plasmids remained detectable (approximately 2 logs decrease in the transfer rate compared with that of pMNI37). The shortest fully active complementing segment (cloned in pMNI40) was deduced as 1,295–2,065 bp of mob_SGI1_. Its 5′‐end lies in the noncoding region (NCR) between *mpsA* and ORF S021 and the 3′‐end is 184 bp upstream of the START codon of S022 in the NCR between S022 and S023.

### 2.3 The role of putative promoters located upstream of ORFs S021 and S022 in SGI1 mobilization

The NCRs preceding ORFs S021 and S022 may contain several promoter‐like sequences, which can drive the expression of the putative RNA factor and we supposed that the lack of these motifs in the dysfunctional complementing plasmids led to the imperfect or failed complementation (Figure 3). The previous results, however, gave no indications on which DNA strand can code for the hypothetical RNA. Therefore, promoters were sought near both ends of the minimal complementing mob_SGI1_ fragment. Two promoter‐like motifs directed toward ORF S021 were found in the NCR upstream of S021 (marked as P1 and P2 in Figure 3a). Removal of P1 had no impact on complementation (compare pMNI34 and pMNI71), whereas the additional deletion affecting the −35 box of the putative P2 promoter prevented the transfer of both SGI1 and the complementing plasmid pMNI36. To test whether the partial deletion of P2 abolished its promoter activity leading to the transfer deficiency, pMNI36 was supplemented with the strong P*_cat_* promoter. The fact that P*_cat_* could not restore the transfer of either SGI1 or the plasmid (compare pMNI36 and pJKI1125, Figure 3a) suggested that the negative effect of this deletion is based on its destructive effect on the RNA factor rather than on impaired transcription due to the lack of the −35 promoter box. This also indicated that the true promoter of the RNA is possibly located on the other strand in the NCR upstream of S022.

This region, where four promoter‐like elements were predicted (P3–P6, Figure 3b) was examined in a modified experimental setup. The complementation assay shown in Figure 3a implied that the transfer rate of the complementing plasmids itself was informative on their ability to produce the RNA transfer factor, and SGI1 was necessary only as the source of MpsAB proteins. Thus, a refined test system was applied for mapping the functional promoters in the upstream region of S022. In the mating assay, R55^ΔTn^ *^6187^* was the helper plasmid, MpsAB proteins were supplied by an expression plasmid that, unlike SGI1, was not mobilizable in the absence of *oriT*, and the transfer rate of different test plasmids was monitored. A series of test plasmids was constructed where the *mpsAB*‐deleted mob_SGI1_ fragment was gradually shortened in the upstream region of S022. If a reduced transfer rate was observed, the P*_cat_* promoter was inserted into the respective plasmid construct to examine whether the extraneous promoter can restore or at least increase the transfer rate. The first constructs were designed to analyze the role of the predicted P3–P6 promoter‐like elements (Figure 3b). As expected, pMNI34, which contained all four promoters and efficiently complemented SGI1‐C^KOS021^ mutant in the previous assay, proved to be transferable with high frequency. A similar transfer rate was observed when the two distal promoter‐like elements P3 and P4 were deleted (pMNI37). In contrast, removal of the next promoter motif P5 caused almost 2 logs to decrease in the transfer rate, which could be fully restored by insertion of P*_cat_* (compare pMNI37, pMNI30, and pMNI46, Figure 3b). These findings suggested that P5 is required for the proper expression of the putative RNA factor, whereas P3 and P4 are not. Deletion of the last promoter‐like element, P6 resulted in a further approximately fivefold drop in the transfer rate. In this case, the insertion of P*_cat_* brought about a 36‐fold increase (pMNI32/pMNI53, Figure 3b), but the transfer frequency did not achieve the level observed with pMNI37 or pMNI46. The next two deletions (pMNI31 and pMNI39) did not cause an additional change in the transfer rate compared with that of pMNI32; however, insertion of P*_cat_* increased the frequency 30‐fold in the case of pMNI31/pMNI52 and had no effect in the case of pMNI39/pMNI47 (Figure 3b). The longest deletion removing the entire sequence downstream of *oriT* caused a further sixfold reduction in the transfer rate, which could not be increased by P*_cat_* (pMNI42/pMNI48). Finally, the negative control plasmid pMNI41, which contained only the full‐length *oriT*_SGI1_, was not transferable. This confirmed again that MpsAB proteins along with the transfer apparatus of the helper plasmid are not enough for mobilization of *oriT*_SGI1_ and implied that the most important functional part of the required transfer factor is encoded in the region between *mpsA* and *oriT*. The results showed that the RNA factor is expressed mainly from P5 promoter, although P6 may also have a minor role. The 5′‐end of the fully active RNA lies in the sequence corresponding to the 1,966–2,012 bp region of mob_SGI1_; however, significant activity remains even after removing the large 5′ part of the RNA corresponding to the region between the promoter and *oriT*. In contrast, deletions affecting the sequences around the START of ORF S021 (see pMNI36 in Figure 3a and S021 KO mutation in Figure 2a–c) completely abolished the transfer that could not be restored by insertion of an extraneous promoter (see pJKI1125 in Figure 3a).

### 2.4 Analysis of the promoters in the upstream region of ORF S022

**FIGURE 4:**
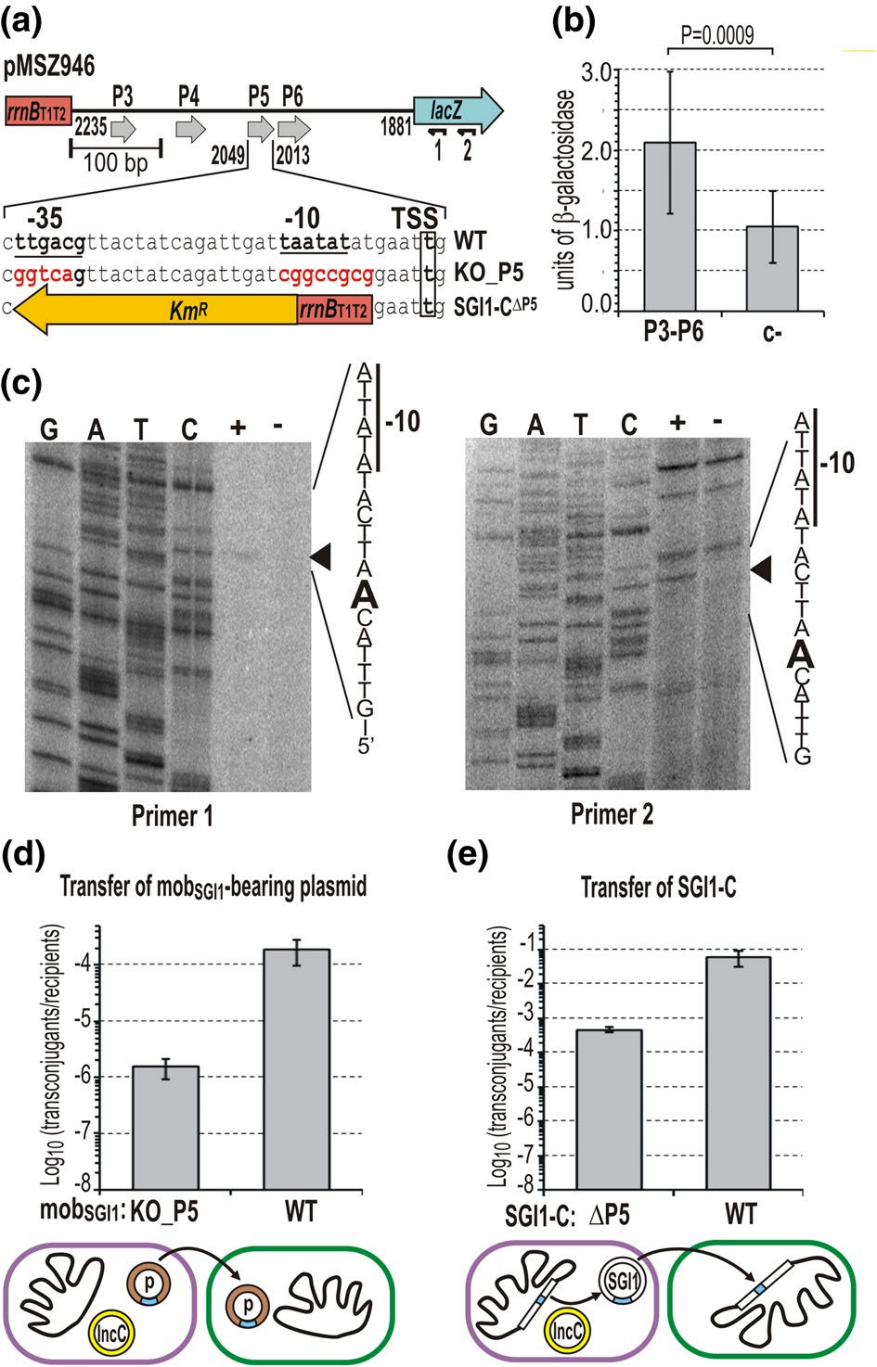
Analysis of promoters located upstream of ORF S022. (a) Schematic map of the upstream region of ORF S022 cloned in the β‐gal test plasmid pMSZ946. The predicted promoters are shown as gray arrows. The −35 and −10 boxes and the TSS of P5 identified by primer extension assay (see Panel (c)) are indicated below the map (WT). The base changes in the KO_P5 promoter mutant version of pMSZ946 are highlighted in red (pMNI11). The replacement by the Km^R^::*rrnB* cassette in SGI1‐C^ΔP5^ mutant is shown below. Oligonucleotides used for primer extension assay are indicated by small arrows marked as 1 and 2. (b) The promoter activity of the upstream region of S022. The activity of P3–P6 promoters was measured by β‐galactosidase assay using pMSZ946. As a negative control (c‐), the empty plasmid pJKI990 (lacking the P3–P6 region) was applied. The bars represent the mean and standard error obtained from nine independent parallels (*n* = 9). (c) Primer extension assay for determination of TSSs in the upstream region of S022. The assay was carried out using two different primers (Primer1: lacZoutE; Primer2: pUCfor24 marked as 1 and 2 in Panel (a), respectively). Lanes G, A, T, C: Sanger sequencing reactions obtained with primers 1 and 2, and the tester plasmid pMSZ946 as a template DNA. Arrowheads point to the A base on the nontranscribed strand corresponding to the TSS on the sense strand. Lane +, pMSZ946, Lane –, pJKI990 (c–). (d) Transfer of the KO_P5 mutant mob_SGI1_‐bearing plasmid. The transfer frequency of P5 promoter mutant plasmid pMNI11 that had the −35 and −10 boxes replaced (see sequences highlighted in red in Panel (a)) was compared with the wt parental plasmid pMSZ949 (WT). (e) Transfer of the promoter mutant SGI1‐C^ΔP5^. The transfer frequency of the SGI1‐C^ΔP5^ mutant in which P5 promoter was replaced with the *rrnB*T1T2::Km^R^ cassette (see Panel (a)) was compared with that of wt SGI1‐C. In the mobilization assays (Panels (d) and (e)), *E. coli* TG90 (Tc^R^) was used as a recipient, whereas the donor strain was TG1Nal/R55^ΔTn^ *^6187^* (Nal^R^Sul^R^Cm^R^Flo^R^) carrying the promoter mutant/wt mob_SGI1_‐bearing plasmid (Km^R^) or SGI1‐C (Sm^R^Sp^R^Sul^R^), respectively. Transconjugants were selected on TcKm or TcSp plates, respectively, explain the experimental setup. The measured transfer events are indicated by arrows on the pictograms below the graphs. Symbols are as in Figure 2. For transconjugant/donor frequency data, see Figure S3

For the identification of active promoters in the NCR upstream of ORF S022, the whole segment (1,881–2,235 bp of mob_SGI1_) was inserted into a β‐galactosidase test plasmid pMSZ946 (Figure 4a). In this construct, the ATG codon of *lacZ* gene was placed in‐frame at the START codon of S022 and the *rrnB*_T1T2_ terminators were inserted at the other end of the NCR to prevent transcription from outer promoters. The β‐gal assay using this plasmid showed a very low promoter activity in this region (Figure 4b). In order to identify the active promoter, a primer extension experiment was carried out using two oligonucleotide primers. Both assays consistently indicated a TSS at the 2,014 bp position, which confirmed the activity of the predicted promoter P5 (Figure 4c). No other TSS was detectable by this method.

To assess the impact of P5 promoter on the transfer, it was knocked out in a mob_SGI1_‐containing test plasmid and a chromosomally integrated SGI1‐C, and both were examined in a mating assay as previously. In the test plasmid, the predicted −10 and −35 boxes of P5 were substituted with extraneous sequences not resembling the consensus σ^70^ promoter motifs (KO_P5), while in SGI1‐C, the entire P5 was replaced with a cassette containing *rrnB*_T1T2_ terminators and the Km^R^ gene (SGI1‐C^ΔP5^) (Figure 4a). The transfer frequency dropped by two orders of magnitude in both cases (Figure 4d,e). The reduced, but well‐detectable residual transfer activity indicated that lower level of transcription occurred from secondary promoters. This result was in agreement with the previous observation that deletion of the distal part of the NCR including P5 promoter (pMNI30, Figure 3b) has a strong negative effect, but it does not terminate the transfer probably due to the presence of weaker promoters in this region (e.g., P6).

### 2.5 Characterization of RNA transcripts synthesized from the promoters in the upstream region of ORF S022

**FIGURE 5:**
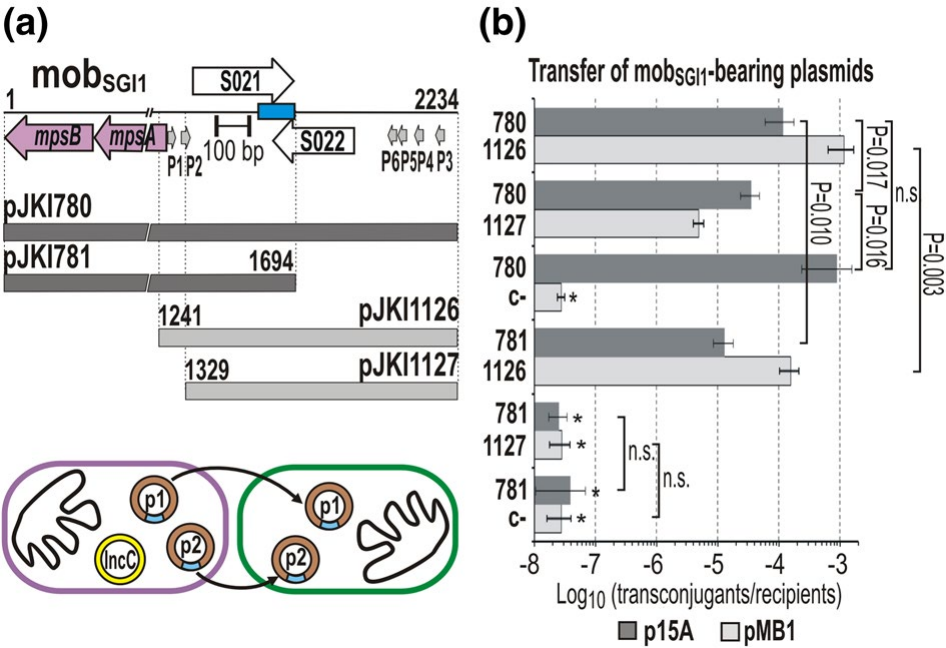
Transcomplementation of different RNA transcripts synthesized from mob_SGI1_ fragments. (a) Description of the plasmids used in the transcomplementation assay. The p15A‐based plasmids (dark gray bars) contain the intact *mpsAB* operon with or without the 3′‐end of mob_SGI1_. pJKI780 containing the full‐length mob_SGI1_ was used as a positive control, whereas pJKI781 has a 3′ truncated version of mob_SGI1_. The pMB1‐based complementing plasmids (light gray bars) carry intact 3′ end of mob_SGI1_ including the P3–P6 promoter region, but pJKI1126 and pJKI1127 contain 5′ truncated mob_SGI1_ fragments (corresponding to that of pMNI34 and pMNI36, respectively, Figure 3a). The pictogram below the graph shows the experimental setup (p1—p15A‐based plasmid, p2—pMB1‐based plasmid). The measured transfer events are indicated by arrows. Symbols are as in Figures 2 and 3. (b) Transfer frequencies of the p15A‐ and pMB1‐based complementing plasmids. The transcomplementation assay was carried out using different pairs of the plasmids shown in Panel (a). In the mobilization assays, *E. coli* TG2 (Tc^R^) was used as the recipient, whereas the donor strain was TG1Nal/R55^ΔTn^ *^6187^* (Nal^R^Sul^R^Cm^R^Flo^R^) carrying the appropriate combinations of the p15A (Sp^R^) and pMB1 (Km^R^Ap^R^) complementing plasmids. Transconjugants were selected on TcSp and TcKm plates, respectively. The pMB1 plasmid backbone without SGI1 sequence (pJKI332) was used as a negative control (c−). The transfer rate of the p15A‐ and pMB1‐based plasmids are indicated as dark and light gray bars, respectively (the numbers refer to the plasmid names shown in Panel (a)). *Transfer frequency was below the detection limit, regular transconjugants were not observed (85% of the few transconjugant colonies carried Km^R^Sp^R^ cointegrates of the test plasmids, the remaining 15% was Cm^R^Km^R^ or Cm^R^Sp^R^ suggesting that these colonies derived from conduction by the helper plasmid). For transconjugant/donor frequency data, see Figure S4

The data accumulated so far not only excluded the participation of proteins in the establishment of the phenotype of S021 KO mutant but also explicitly suggested the existence of an RNA factor whose expression occurs mainly from P5 promoter located upstream of S022 (Figures 2 and 3d,e). The deletion analyses (Figure 3a,b) showed that the essential core domain of this hypothetical RNA is encoded in the region of the 5′‐end of S021, at least 570 bp from the TSS of P5 promoter, suggesting an unusually long RNA compared with known sRNAs involved in different control mechanisms in bacteria. To assess whether a single long RNA or several short RNAs are responsible for the observed phenotype, a two‐plasmid complementation assay was carried out. The p15A‐based test plasmids contained the entire mob_SGI1_ (pJKI780, wt control) or its 3′ truncated derivative lacking the downstream part of mob_SGI1_ from the 3′‐end of *oriT* (pJKI781). Both plasmids produced MpsAB proteins and contained intact ORF S021 (including the *oriT*), but in pJKI781, the P3–P6 region, the identified TSS and the sequence encoding the 5′‐end of the putative RNA was absent (Figure 5a). The pMB1‐based complementing plasmids in turn carried the 3′‐end of the mob_SGI1_ region without *mpsAB* and expressed the fully functional (wt control) or a truncated nonfunctional version of the presumptive RNA. The wt control pJKI1126 carried the mob_SGI1_ fragment identical to that of pMNI34, which proved previously transferable and could complement SGI1‐C^KOS021^, whereas pJKI1127 contained the slightly shorter fragment present in pMNI36, which was inactive in transfer and complementation (Figure 3a). The empty plasmid vector without any mob_SGI1_ sequence was applied as a negative control (c−). Every combination of the compatible test plasmids was introduced into the donor strain TG1Nal/R55^ΔTn^ *^6187^* and their transfer rate was measured in a mating assay. All plasmids (except c−) carried intact *oriT*, therefore, their transfer was detectable in the same mating. As expected, both plasmid partners were transferable (except c−), if the full‐length mob_SGI1_ was present in the p15A‐based plasmid pJKI780 (this part of the assay can be regarded as a positive control). The maximal transfer rate for pJKI780 was obtained in the presence of the nonmobilizable negative control (c−). This rate was slightly (but not significantly) reduced when pJKI780 was paired with pJKI1126 even though it could produce functional RNA. This observation can be explained by the competition between the two plasmids for MpsAB proteins and other components of the transfer apparatus that are provided by the low‐copy plasmid pJKI780 and the single‐copy helper plasmid, respectively. Titration of the mobilization proteins by *oriT* of the high‐copy plasmid leads to reduced levels of these components available for the low‐copy partner. Further decrease of pJKI780 transfer occurred when the high‐copy partner carried the 5′‐truncated nonfunctional mob_SGI1_ fragment (pJKI1127). In this case, competition exists not only for MpsAB and the transfer apparatus but also for the functional RNA produced only by the low‐copy pJKI780. The negative effect of the functional RNA deprivation was more obvious if the transfer of the high‐copy plasmids were compared: in the presence of pJKI780, the transfer rate of pJKI1127 producing the 3′‐truncated (possibly nonfunctional) RNA was about two orders of magnitude lower than that of pJKI1126 (Figure 5b). On the other hand, the 3′‐truncated mob_SGI1_ (pJKI781) could only be mobilized if the longer, functional RNA was expressed by the high‐copy plasmid partner (pJKI1126). The transfer rate of pJKI781, however, did not reach that of wt mob_SGI1_‐bearing counterpart (compare pJKI780 and pJKI781 when complemented by pJKI1126, Figure 5b). No complementation was observed when both plasmid partners contained the nonfunctional version of the mob_SGI1_ fragments (pJKI781 + pJKI1127). These results indicated that deletions in the P5 promoter region and in the surroundings of the 5′‐end of S021 affect the same long RNA.

**FIGURE 6:**
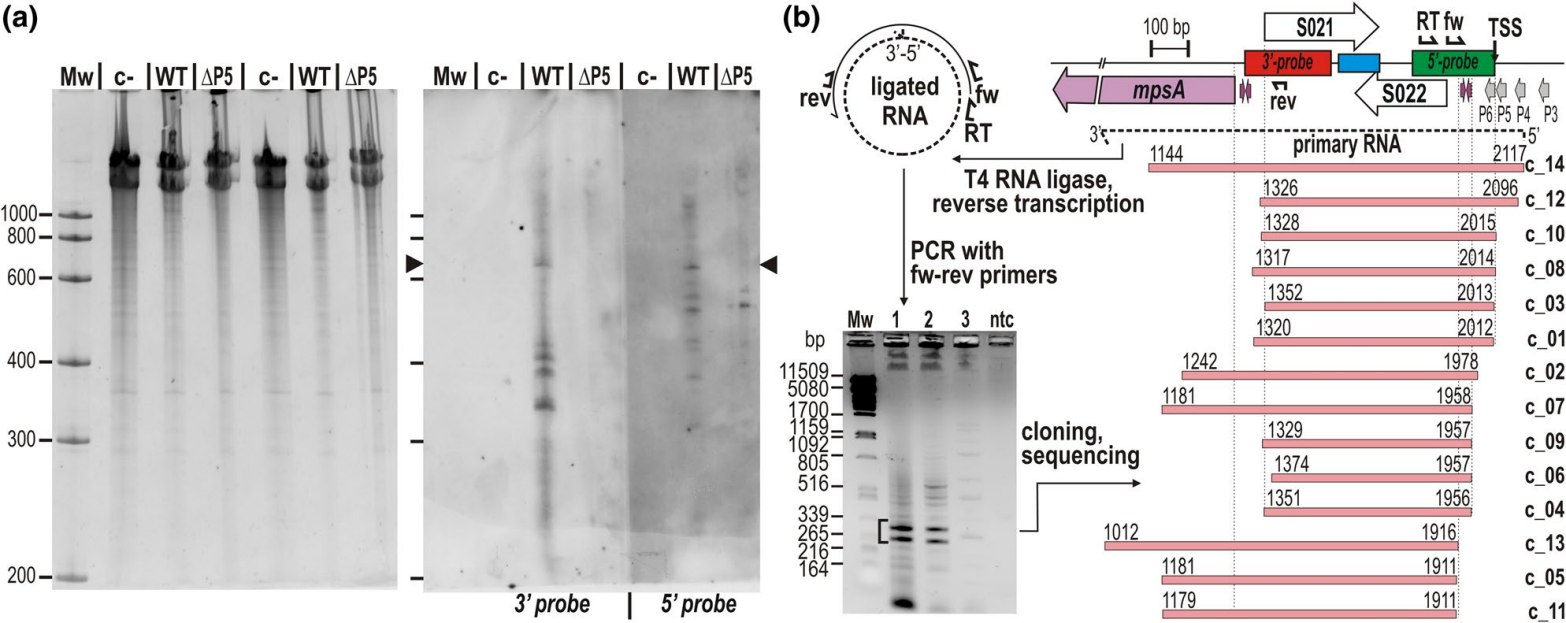
Detection of RNA transcripts synthesized from P5 promoter. (a) Northern hybridization assay. Total RNA was extracted from strain TG1Nal::*repA*_SGI1_ containing wt or ΔP5 mutant SGI1‐C. The original strain without SGI1 was used as a negative control (c−). The RNA samples were separated on 5% denaturing TBE‐polyacrylamide gel (left‐side panel) and then blotted onto a nylon membrane (right‐side panel). The samples in Lanes 2–4 and 5–7 are c−, WT, and ΔP5. The two halves of the blot were hybridized with the 3′‐ and 5′‐probes whose length and position are indicated as red and green boxes (see the map in Panel (b)). Arrowheads point to the 650–700‐base‐long primary RNA transcript hybridized to both probes. (b) Determination of the 5′‐ and 3′‐ends of the primary transcripts initiated by promoters upstream of S022. The cartoon describes the major steps of the analysis. After the circularization of RNAs by T4 RNA ligase, reverse transcription, and subsequent PCR amplification of the 3′‐5′ joint fragments was carried out using the primers: RT, S022promseq; fw, S022promfor_Nc; rev, SGI1_S021promseq (see Table S3). The gel image shows the results of PCRs with fw‐rev primers enclosing the ligation site. Templates were: cc and 10× diluted reaction mixture obtained from reverse transcription (Lanes 1 and 2, respectively); ligated RNA sample before reverse transcription (Lane 3); ntc: nontemplate control; Mw: λ DNA digested with PstI. Fragments excised for cloning are indicated by a bracket. The TSS identified by primer extension, and the location and orientation of the primers used are shown in the map of the region. The terminator‐like inverted repeats are indicated by purple arrows. Pink bars with coordinates represent the RNA species deduced from the sequences of the cloned 3′–5′ junction fragments (c_01–c_14)

Therefore, the RNA species synthesized from P5 promoter region were analyzed by Northern hybridization, where the chromosomal SGI1‐C^WT^ and the promoter mutant SGI1‐C^ΔP5^ were compared. One of the probes was complementary to the 5′‐end of RNAs and hybridized near the TSS (5′‐probe), whereas the other overlapped the region affected by the KO S021 mutation and hybridized to RNAs that include sequences located at 470–720 bp from the TSS (3′‐probe, Figure 5a). The hybridization gave negative results when the total RNA was isolated from strains that carried a single chromosomal copy of SGI1‐C (data not shown), therefore both the wt and ΔP5 mutant were transferred into the strain expressing the RepA protein of SGI1 (Szabó et al., 2021) to increase the copy number of the island. Northern analysis using the two probes (Figure 6a) showed that the total RNA sample extracted from the strain carrying SGI1‐C^WT^contained several hybridizing RNA species of different lengths, whereas the sample from ΔP5 mutant did not (only few weak bands were detectable with the 5′‐probe, which might be synthesized from P6 promoter), confirming that the elimination of P5 promoter significantly decreased the transcription from this region. A 650–700‐base long RNA was detectable with both probes in the wt sample, supporting our results from the trans‐complementation test (Figure 5). Based on its estimated length, the 3′‐end of this transcript lies close to the START of ORF S021. The presence of shorter RNAs detectable with the 3′‐probe suggested that the primary transcript is posttranscriptionally processed, which results in smaller RNA species consisting the 3′‐end of the primary transcript. On the other hand, the shorter RNAs detected by the 5′‐probe may derive from the activity of several terminator‐like motifs causing earlier interruption of transcription. The most efficient termination occurred 70–80 base downstream of the TSS, where the vast majority of transcription was stalled (Figure S5). The 11‐bp imperfect inverted repeat located near this site (1,923–1,948 bp of mob_SGI1_) can form a hair‐pin structure on the RNA and may be responsible for this effect.

To determine the 5′ and 3′ termini of the long primary transcripts, RT‐PCR analysis was carried out using 5′‐3′‐end‐ligated RNA as a template (Kuhn & Binder, 2002). Total RNA was isolated from the TG1Nal::*repA*_SGI1_/SGI1‐C^WT^ strain, and after joining the 5′‐ and 3′‐ends by T4 RNA ligase the circularized RNA was used as a template for reverse transcription. Then, the 5′–3′‐end junctions were amplified from the cDNA population with primers enclosing the ligation site (Figure 6b). Sequencing of the cloned amplicons revealed that a heterogeneous RNA population is synthesized from the region of interest as suggested by the Northern analysis. The majority of the RNAs were terminated near the START site of S021 and their length correlated with the approximately 700 bp band seen on the Northern blot, while several clones represented RNAs that ended in the *mpsA* gene. Although the primer extension experiment detected only one TSS (belonging to P5 promoter), this assay suggested the existence of several alternative start sites. The 5′‐end of four RNAs (clones c_01, c_03, c_08, and c_10, Figure 6b) was at or near the position of TSS of P5 (2,014 bp of mob_SGI1_ or +1, −1, −2 base difference). T4 RNA ligase can circularize only uncapped RNAs and the lack of 5′‐pyrophosphate cap makes RNA sensitive for degradation, which may explain the divergence observed in the 5′‐ends. The similar variance was detected in the other four clones, which started around the 1,957 bp position (c_04, c_06, c_07, and c_09). The next group of transcripts started at or near 1,911 bp (c_05, c_11, and c_13). The −10 box of the nearest predicted promoter P6 is located at 1,986–1,993 bp, thus it seems impossible that it could initiate transcription at a distance of 30 and 75 bp downstream. Even though the sequence of these clones supported the existence of two additional TSSs, promoters could not be found by in silico methods at the appropriate positions. Thus, these putative “TSSs” are formed rather by cleavage of longer transcripts at specific sites susceptible to the attack of endo‐RNases. Interestingly, two RNAs started upstream of the P5 TSS suggesting that weak upstream promoters could also initiate transcription with lower frequency. Nevertheless, the results clearly indicated that many transcripts initiated in the upstream region of S022 terminate in the critical region surrounding the START of ORF S021. These results also suggested that the primary transcripts undergo maturation steps, by which the biologically active transfer factor, called sgm‐sRNA (*SG*I1 *m*obilization *s*mall RNA), may be formed. It is worthy of note that the long primary RNA transcripts include the entire S022 ORF, which has an unambiguous SD‐box making its translation possible. Consequently, these RNAs can also serve as mRNAs for S022. Although the production and function of S022 protein were not directly examined, the involvement of this protein in SGI1 transfer was excluded (Kiss et al., 2019).

### 2.6 Mapping of the core functional part of sgm‐sRNA

**FIGURE 7:**
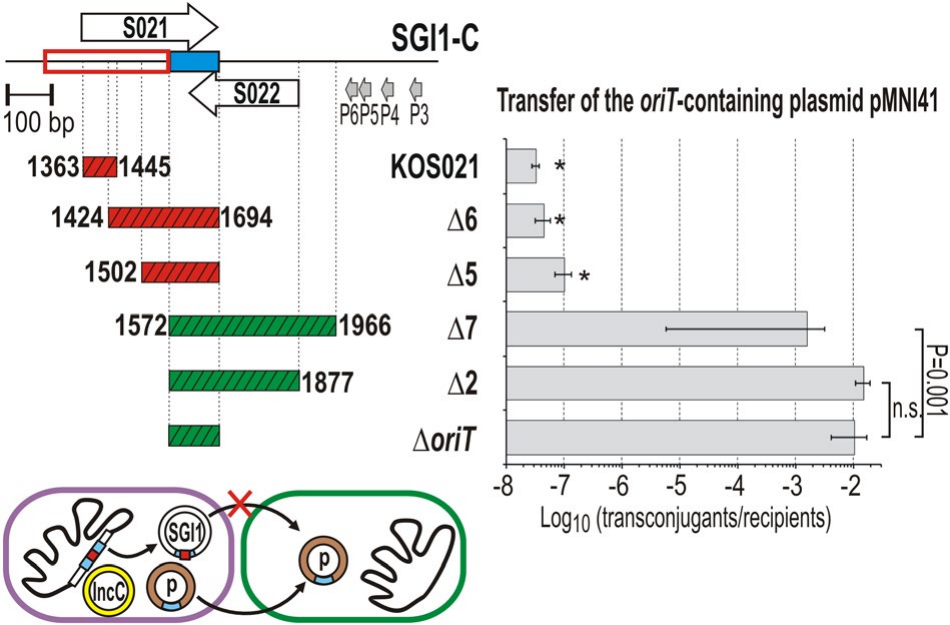
Mapping of the core functional part of sgm‐sRNA. The mobilization of pMNI41 carrying the *oriT*_SGI1_ was measured in the presence of R55^ΔTn^ *^6187^* helper and different SGI1‐C deletion mutants (see pictogram below the graph). The deletions Δ2–Δ7 affect the part of mob_SGI1_ region that encodes sgm‐sRNA. Note that these SGI1‐C deletion mutants are not mobilizable in the absence of *oriT* (indicated by the red box on the pictogram) but provide the SGI1‐encoded factors for mobilization of the *oriT*‐bearing plasmid, pMNI41 (transcomplementation). SGI1‐C^KOS021^ and SGI1‐C^Δ^ *^oriT^* were used as negative and positive controls, respectively. The hatched horizontal bars represent the deleted parts of mob_SGI1_ in the complementing SGI1‐C, green—mobilization of pMNI41 occurs; red—plasmid transfer is not detectable. The deduced core domain of sgm‐sRNA is indicated as a red rectangle. In the mobilization assays, *E. coli* TG2 (Tc^R^) was the recipient, while the donor strain was TG1Nal/R55^ΔTn^ *^6187^* +pMNI41 (Nal^R^Cm^R^Flo^R^Km^R^) carrying one of the different SGI1‐C deletion mutants (Sm^R^Sp^R^). The pMNI41 transconjugants were selected on TcKm plates. *Transfer frequency of pMNI41 was below the detection limit. SGI1‐C transfer was also undetectable (<2.7–9.0 × 10^–8^/recipients) in each mating. For transconjugant/donor frequency data, see Figure S6

The deletion analyses showed that 3′‐end of the functional sgm‐sRNA species lies in the noncoding region between *mpsA* and S021 (Figure 3a), while the 5′‐end was not localized into a similarly narrow region (Figure 3b). Previous results proved that longer DNA segments, such as the entire *oriT* or the 5′‐end of S022 (Δ*oriT* and KO S022 mutations, respectively), can be deleted from or replaced in the sgm‐sRNA without negative effects on SGI1 transfer (Kiss et al., 2019). To determine the core functional part of sgm‐sRNA, inner deletions were generated in a chromosomal SGI1‐C between the START of S021 and the promoter region of S022 (Figure 7). Since the deletions left the entire P3–P6 region intact, these SGI1‐C mutants possibly expressed deletion derivatives of the primary transcript similarly to the wt island. The functionality of these derivatives, that is, their ability to mature to active sgm‐sRNA, was examined in a complementation experiment, where the transfer of the *oriT*‐bearing plasmid, pMNI41, was monitored in the presence of R55^ΔTn^ *^6187^* and one of the SGI1‐C deletion mutants. In this experimental setup, SGI1‐C mutants supplied MpsA, MpsB, and the sgm‐sRNA for pMNI41 transfer, but they were not mobilizable due to the lack of *oriT*, which was removed by the deletions.

Similar to the KO S021 mutation, the deletions in SGI1‐C between the START of S021 and *oriT* (Δ5, Δ6) completely abolished pMNI41 transfer. In contrast, deletions directed from *oriT* toward the TSS did not, suggesting that the same sgm‐sRNA remained functional in these cases. SGI1‐C^Δ7^ containing the largest deletion, which removed the *oriT*, the entire S022, and its 5′‐UTR, was also active in complementation. However, it was about one to three orders of magnitude and less efficient compared with that of SGI1‐C^Δ^ *^oriT^* or SGI1‐C^Δ2^ (pMNI41 mobilization by SGI1‐C^Δ7^ mutant showed high standard deviation in three independent experiments, each including six biologic replicates). Considering these and the previous results (Figures 7 and 3a), we concluded that the region indispensable for the function of sgm‐sRNA begins in the 1,502–1,572 bp part of mob_SGI1_ and terminates at the 1,295–1,329 bp region. It is worth noting that the *oriT* sequence appeared not to be part of the core functional domain of sgm‐sRNA (Figure 7).

## 3 DISCUSSION

SGI1‐family elements are sophisticated parasites of IncA and IncC plasmids as they do not simply utilize the plasmid‐encoded T4SS but exploit the control mechanisms of the helper plasmid for timing of their excision and plasmid‐like replication (Kiss et al., 2015; Szabó et al., 2021). They modify the T4SS for their own benefit (Carraro et al., 2017) and destabilize the helper (Durand et al., 2021b; Harmer et al., 2016; Szabó et al., 2021), possibly in order to ensure the stable vertical transfer. These interventions increase the efficiency of SGI1 transfer and the stability of the island in the host bacteria when coexisting with the helper plasmid. SGI1 encodes an FlhDC‐family activator and three T4SS components that are related to and can substitute their helper‐encoded homologues. These SGI1 derived proteins have important roles in the parasitism of SGI1 on the helper plasmid (Carraro et al., 2017; Durand et al., 2021b).

In contrast, the genes clustered in the 2.2 kb mob_SGI1_ module are unrelated to the genes of the conjugation apparatus of IncC plasmids and the sequence and organization of *oriT*_SGI1_ do not resemble the *oriT* of IncC plasmids (Kiss et al., 2019). MpsA and MpsB, the two mob_SGI1_‐encoded proteins essential for SGI1 mobilization are related to the recombinases of the λ integrase family and not to relaxases. MpsA, as a first example among IEs, has been suggested to act as an atypical relaxase of SGI1‐family elements, similar to TcpM and MobK, the Tyr‐recombinase‐related relaxase proteins of plasmids pCW3 and pIGRK, respectively (Nowak et al., 2021; Wisniewski et al., 2016). The other two putative proteins encoded by ORFs S021 and S022 appear unrelated to any known proteins. Whereas S022 transcript is present at a relatively high quantity compared with those of most backbone genes, the level of S021 transcript appears the lowest among SGI1 ORFs (Golding et al., 2007). While ORF S022 is preceded by an ideal SD‐box suggesting the translation of S022, the ORF S021 has no obvious ribosome binding site, thus very small amount of the putative S021 protein can be synthesized if it is expressed at all. As both putative proteins appear to be unnecessary for SGI1 transfer, until now, mob_SGI1_ has been regarded to express only two transfer factors, MpsA and MpsB.

In this work, we report the identification of a new factor indispensable for SGI1 transfer, which proved to be an RNA molecule named sgm‐sRNA. The existence of an additional soluble transfer factor was discovered during the phenotype analysis of S021 KO mutant SGI1‐C, which was not mobilizable, but its transfer deficiency could be rescued by the mob_SGI1_ region in trans (Figure 2b–d). The S021 KO mutation was designed to inactivate the S021 gene, however, expression of S021 protein in trans could not complement the SGI1‐C^KOS021^ mutant (Figure 2d). On the other hand, the frameshift mutations **fs1–4**, which also disrupted ORF S021 and the other overlapping short ORFs, did not cause transfer deficiency of the mob_SGI1_‐containing plasmids (Figure 2e). These observations suggested that the transfer factor expressed in the wt mob_SGI1_ but not in SGI1‐C^KOS021^ cannot be a protein.

The complementation studies of SGI1‐C^KOS021^ and the transfer assays carried out with plasmids containing gradually shortened mob_SGI1_ fragments indicated that the 1,295–2,065 bp region of mob_SGI1_ is as efficient in complementation as the full‐length mob_SGI1_ (Figure 3a). This implied that the minimum length of the presumed RNA, or at least its primary form, is 700–750 base long, but gave no indication on which strand the sgm‐sRNA is encoded. Promoter predictions revealed the presence of several promoter‐like motifs near both ends of this region. Partial deletion of the putative promoter P2 preceding ORF S021 completely abolished the complementation of SGI1‐C^KOS021^, but this could not be restored by substitution with P*_cat_*. On the other hand, deletion of P5 and P6 localized upstream of ORF S022 on the complementary strand caused a less dramatic decrease of transfer and P*_cat_* could rescue these deletions (Figure 3b). These results showed that the functional sgm‐sRNA is synthesized from a promoter located in the NCR between S022 and S023. Interestingly, removal of the entire downstream part of mob_SGI1_ from *oriT* to the promoters P3–P6 did not fully terminate the transfer, indicating that the functional part of sgm‐sRNA is located in the distal part (3′‐end) of the primary transcript. Mobilization of the *oriT*‐bearing pMNI41 by the inner deletion mutants of SGI1‐C (Δ2, Δ5–7, Δ*oriT*, and KOS021, Figure 7) also showed that removal of the 1,572–1,966 bp of mob region proximal to promoters P3–P6 is not deleterious for the transfer, while all deletions affecting the distal region (1,363–1,572 bp) completely terminate it.

The sequence of P5 promoter (ttgacg‐16 bp‐taatat) shows the best match to the σ^70^ consensus, suggesting that P5 is the strongest one in the vicinity of the four predicted promoters. It was supported by the fact that only one TSS, preceding P5, was detectable by primer extension analysis (Figure 4c). This was also consistent with the reduced transfer frequencies observed with the KO_P5 mutant plasmid pMNI11 and SGI1‐C^ΔP5^ (Figure 4d,e), and the result of Northern analysis, where no RNA was detectable in case of SGI1‐C^ΔP5^ mutant (Figures 6a and S5). Further support was obtained from the RNA ligation experiment, where the start point of four RNAs was very close to the TSS of P5 (Figure 6b). Thus, we concluded that the RNA transcript required for SGI1 transfer is synthesized mostly from P5 promoter.

The transcomplementation experiment carried out with two halves of mob_SGI1_—that are per se not transferable—indicated that the deletion mutations affecting the 5′ and 3′ parts of mob_SGI1_ are in linkage, which anticipated an RNA spanning the downstream part of mob_SGI1_ (e.g., from the NCR between *mpsA* and S021 to the promoter region upstream of S022, Figure 5a,b). Northern hybridization with probes complementary to the 5′ and 3′ parts of the presumed RNA confirmed the presence of a 650–700 base long RNA in the total RNA extract isolated from an SGI1‐C^WT^‐bearing host strain, whereas the same RNA was missing in the case of SGI1‐C^ΔP5^ (Figure 6a). This long RNA spans the entire S022–S021 region and possibly terminates near the 9‐bp imperfect inverted repeat (IR) located upstream of *mpsA* (1,283–1,309 bp of mob_SGI1_). This sequence motif can form a stem‐loop structure on the RNA and may act as a termination signal or a cleavage site for endo‐RNases (Bechhofer & Deutscher, 2019). In the RNA‐ligation experiment, the majority of cloned 3′–5′ junctions derived from RNAs whose 3′‐end was at a distance of 8–43 base to this motif, which may support the latter possibility. (Figure 6b).

Beyond these transcripts, shorter RNA fragments were also detectable with both probes. The ones hybridized to the 5′‐probe could be formed by early termination or endonucleolytic cleavage by endo‐RNases. The most abundant short RNA (corresponding to the 5′ end of the primary transcript) terminates about 70–80 base from TSS of P5 (Figure S5) and is possibly formed due to the presence of the GC‐rich 11‐bp IR located 66 bp downstream of TSS. This IR motif can form a stem‐loop structure that can act as a transcription terminator or serve as a specific target of RNases, which likely participate in the normal maturation of sgm‐sRNA. The efficient termination or endonucleolytic cleavage at this site can account for the relatively low amount of full‐length RNA (Figures 6a and S5) and the very weak transcription activity measured at the START codon of S022 (Figure 4b), even though P5, at least according to its sequence, appears to be a strong promoter. The fact that deletions between *oriT* and P5 (eliminating the 5′ half of the RNA) have no serious effect on SGI1 transfer (Figure 7) suggests that the ~70 base long RNA is not functional form and is apparently a by‐product. By all means, the terminator‐like IR appears to have a key role in controlling the amount of the full‐length primary transcripts and consequently the functional sgm‐sRNA.

Hybridization with the 5′‐probe revealed the presence of several further RNA species, whose length ranges between approximately 350 and 600 base and appear similar or less abundant than the 650–700 base long primary transcript. These derivatives can be formed also by early termination or site‐specific RNA cleavage causing 3′ truncation of the transcript and leading to the partial or entire removal of the core region required for the activity of sgm‐sRNA. Thus, most of these RNAs are probably not functional. In contrast, the 3′‐probe covering the core domain of sgm‐sRNA (Figures 3 and 7) possibly hybridized to predominantly functional derivatives of the primary transcript. The detection of four different derivatives (ranging between about 450 and 330 base in length) using the 3′‐probe suggests that the maturation of the final form of sgm‐sRNA (and also its elimination) occurs via consecutive cleavage steps. The results obtained from the 5′–3′‐end‐ligation experiment also support this hypothesis. The 5′‐ends found downstream of TSS of P5 are clustered into two groups, where the 5′‐ends lie 56–58 bases or 98–103 bases from the TSS. These RNA start points, however, are not preceded by promoter‐like elements and probably derive from endonucleolytic cleavage.

Surprisingly, 8 of the 14 identified 3′‐ends occur in the 1,317–1,374 bp range. The previous deletion analysis (Figure 3a) showed that the 3′‐end of the functional sgm‐sRNA is located between 1,295 and 1,329 bp of mob region, therefore, most of these RNA species are apparently not functional. The existence of numerous RNAs partially or entirely lacking the core domain of sgm‐sRNA may indicate a rapid turnover of the functional form, which probably derive from transcripts whose length reaches or exceeds the approximately 700 base, that is, they terminate near the position of the hairpin‐like element located adjacent to the START of *mpsA* (Figure 6b).

The world of bacterial small RNAs is amazingly diverse. These transcripts are often short (approximately 50–250 base) noncoding RNAs synthesized in intergenic regions from their own promoters, but many of them are generated from 5′‐ or 3′‐UTRs or even from the coding regions of mRNAs by transcription or posttranscriptional processing (Wagner & Romby, 2015). The vast majority of, if not all, known sRNAs participate in regulatory mechanisms. Lots of them are antisense (as)RNAs and are involved in transcriptional or posttranscriptional control of gene expression (Wagner & Romby, 2015). Small RNAs or countertranscribed (ct)RNAs have been identified as key elements in copy‐number control of many plasmids (Wagner et al., 2002; Wagner & Simons, 1994). Small RNAs can act as antitoxins in TA systems where they can not only control the transcription of a toxin protein as antisense of the mRNA (Type I TA) (Brantl, 2012) but also keep the toxin inactive by direct RNA‐protein binding (Type III) (Blower et al., 2012). Bacterial sRNAs fulfill their regulatory functions in astonishingly diverse ways (Jørgensen et al., 2020; Wagner & Romby, 2015) from the direct competition for the ribosome binding site of the target mRNA, through regulation of translation initiation or transcription termination (Bossi & Figueroa‐Bossi, 2016), to the control of mRNA lifetime by riboswitches (Richards & Belasco, 2021). In many cases sRNAs act by accelerating or delaying the degradation of the target mRNA or other regulatory sRNA by promoting or even impeding RNase E cleavages (Bandyra et al., 2012; Fröhlich et al., 2013). Base pairing of sRNAs and their target RNA often requires the involvement of RNA chaperones like Hfq, ProQ, and CsrA that facilitate the access of sRNAs to their target by binding the RNA partners on their surface (Holmqvist & Rizvanovic, 2020; Quendera et al., 2020). Another group of RNA‐binding proteins is RNases that are responsible for RNA maturation and degradation. RNase E, RNase III, and PNPase appear the most important enzymes in sRNA turnover and processing (Quendera et al., 2020). RNA chaperones along with RNases have a central role in the sRNA‐based global control network (Bechhofer & Deutscher, 2019), (Quendera et al., 2020).

Although sRNAs play important role in the regulation of many different metabolic and transport mechanisms, and other biological functions such as motility, biofilm formation and virulence (Wagner & Romby, 2015), sRNAs involved in the conjugal transfer have rarely been reported. FinP repressor in F‐like plasmids is one of the first discovered asRNA regulators that control conjugative transfer (Finlay et al., 1986). FinP alone can repress *traJ* encoding an activator of *tra* genes but normally acts in concert with the RNA‐binding corepressor, the FinO RNA‐chaperon (Koraimann et al., 1996). More recent examples for sRNAs controlling conjugation are the Anti‐Q transcription attenuator of the *E. faecalis* plasmid pCF10 (Shokeen et al., 2010), RteR repressor of the *tra* operon of the *Bacteroides* ICE CTnDOT (Waters & Salyers, 2012), and RprA, which acts as a translation activator by an anti‐antisense mechanism on many mRNAs, including *ricI* mRNA that encodes for RicI, an inhibitor of the transfer of pSLT virulence plasmid of *Salmonella* (Papenfort et al., 2015).

We have shown that sgm‐sRNA expressed by SGI1 derives from the 3′‐UTR of the transcript initiated from P5 promoter. Its primary transcript can serve as mRNA of ORF S022, although the putative S022 protein is not necessary for SGI1 mobilization. D eletion, Northern, and RNA 5′–3′‐end‐joining analyses indicated that the functional sgm‐sRNA is formed from the 3′‐end (1,295–1,572 bp of mob_SGI1_) of the full‐length primary transcript possibly via posttranscriptional maturation, that is, RNase cleavages (a predicted secondary structure of the core domain is shown in Figure S7). However, it should be noted that the presence of the 5′‐end of the primary transcript can significantly modify the folding of this region. Although the 5′‐end of the primary transcript (1,572–2,012 bp of mob_SGI1_) was not necessary for the function, removal of the 1,877–1,966 bp of mob region caused a decrease in SGI1 transfer (SGI1‐C^Δ7^, Figure 7), and the 5′‐ deletions could not completely be rescued by substitution with the extraneous promoter P*_cat_* (Figure 3b). This implies the importance of this region probably in the correct folding that may be required for the proper maturation of the final sgm‐sRNAs. Surprisingly, the *oriT* sequence, which includes three inverted repeat motifs (Kiss et al., 2019) that can form strong secondary structures in the primary transcript is not included in the core functional part of sgm‐sRNA, indicating that basepairing with *oriT* or recognition of this sequence by proteins in the sRNA is not required for its function.

Comparing 159 SGI1‐family elements found in GenBank, the upstream region of *mpsA* encoding the primary transcript of sgm‐sRNA appears well conserved (Figure S8). The core region of sgm‐sRNA seems similarly conserved as *oriT*, whereas ORF S022 and its upstream region show somewhat greater variability mainly in more distant relatives of SGI1 such as GI*Vch*O27‐1 (CP010812). This is consistent with the importance of sgm‐sRNA in the mobilization of SGI1‐like elements. SGI1‐B2 from *Proteus mirabilis* strain PmSC17 carries a large IS*26*‐based transposon inserted at the proximal end of the core domain of sgm‐sRNA (1,534 bp in mob_SGI1_), for example, between the 1,502 and 1,572 bp positions, where the 5′‐end of sgm‐sRNA was localized (Figure 7). Therefore, the mobilization properties of SGI1‐B2 may indicate whether the insertion interferes the functionality or maturation of sgm‐sRNA.

Based on the modes of action of known sRNAs, one of the most plausible role of sgm‐sRNA would be to activate the expression of *mpsAB* operon. However, several facts seem to be inconsistent with this model. MpsAB proteins were successfully expressed from pJKI882 where the operon is driven by the P_tac_ promoter, or from constructs, where *mpsAB* was preceded by the entire or only 20 bp of the upstream NCR (Kiss et al., 2019). This suggests that translation of *mpsAB* mRNA is not inhibited by secondary structures that would require derepression or activation by the sgm‐sRNA. Furthermore, expression of MpsAB proteins in trans proved insufficient to rescue the S021 KO mutant, indicating that the transfer deficiency of SGI1‐C^KOS021^ is not due to the MpsAB deprivation.

Theoretically, the role of sgm‐sRNA might be the activation of an IncC helper‐encoded gene that is necessary for SGI1 mobilization but not for the plasmid transfer, or the repression of a gene specifically blocking SGI1 mobilization, however, at the moment there are no indications for the existence of such genes.

Another possibility would be that sgm‐sRNA fulfills its function by binding or even recruiting proteins. In case of Type III TA systems, the antitoxin sRNA specifically binds to and inhibits the toxin protein (Blower et al., 2012; Fineran et al., 2009). To the best of our knowledge, there are no reports on the involvement of sRNAs in conjugation complexes, however, this can be a conceivable role for sgm‐sRNA. The fact that the mobilization proteins are completely unrelated to those of the helper IncC plasmids raises the question of how the initiation complex of SGI1 is assembled on *oriT* and transported to T4SS of the helper plasmid. A similar question has been posed for the initiation complex of pIGRK, which is mobilized by the unrelated conjugation system of RP4 (Nowak et al., 2021). It was previously shown that the IncC helper‐encoded relaxase TraI is not necessary for SGI1 mobilization, but increases the transfer rate of the island with orders of magnitude (Kiss et al., 2019). On the other hand, the coupling protein TraD of the helper is obligatory for SGI1 transfer (unpublished results). These facts suggest that both key proteins of the IncC conjugation apparatus should interact somehow with the unrelated transfer proteins MpsA and MpsB, the possible elements of the initiation complex of SGI1. This interaction might occur with the aid of sgm‐sRNA. In this case, sgm‐sRNA would be the first example of a small RNA that is directly involved in the formation or transport of a conjugative complex. Involvement of an sRNA in the transfer of SGI1 reflects that the members of the SGI1‐family apply unusual strategies to ensure their horizontal spread and exceptional evolutionary success.

## 4 EXPERIMENTAL PROCEDURES

### 4.1 Microbial techniques and DNA procedures

Relevant features of the bacterial strains and plasmids are listed in Tables S1 and S2, respectively. Bacterial strains were grown at 37°C in Luria‐Bertani (LB) broth or plates supplemented with the appropriate antibiotics used at a final concentration as follows: ampicillin (Ap) 150 μg/ml, chloramphenicol (Cm) 20 μg/ml, kanamycin (Km) 30 μg/ml, spectinomycin (Sp) 50 μg/ml, streptomycin (Sm) 50 μg/ml, nalidixic acid (Nal) 20 μg/ml, and tetracycline (Tc) 10 μg/ml. Plasmids with temperature‐sensitive pSC101 replication system were maintained and cured at 30v and 42°C, respectively.

The Sm^R^/Sp^R^Sul^R^ SGI1‐C variant used in this work is the spontaneous deletion derivative of a wt SGI1 identified in a Hungarian *S*. Typhimurium DT104 isolate named as strain ST1375 (Kiss et al., 2012). The genome sequence of ST1375 (unpublished) proved that its chromosomal SGI1 is identical to the published SGI1 sequences found in other *S*. Typhimurium isolates (GenBank numbers: CP014358, CP012985, CP007581, CP014969, CP014967, and HF937208). The Sm^R^/Sp^R^Sul^R^Ap^S^Cm^S^Flo^S^Tc^S^ segregant of ST1375, named ST21S/1 emerged during a passage experiment (Kiss et al., 2012). Strain ST21S/1 carries an SGI1 derivative that is identical to the known SGI1‐C variant described by (Boyd et al., 2002) and has been shown to have the same mobilization properties as the parental SGI1 (Kiss et al., 2012). This SGI1‐C was mobilized by R55 into *E. coli* TG1Nal strain from ST21S/1 resulting in strain TG1Nal::SGI1‐C^WT^ (Kiss et al., 2015), which was used in mating assays, mutagenesis and applied as a template in PCRs for cloning.

Standard molecular biology procedures were carried out according to Sambrook et al. (1989). *E. coli* TG1 strain was used for cloning work except in cases of R6Kγ‐based replicons, which were maintained in S17‐1 λpir (Simon et al., 1983). Detailed methodology of plasmid constructions is described in Supplementary Methods. Test/colony PCRs were performed using Dream Taq polymerase (Thermo Fisher Scientific) as described (Kiss et al., 2012). DNA fragments of SGI1‐C were amplified for cloning with Phusion (Thermo Fisher Scientific) or Pwo (Roche) polymerases and sequenced on ABI Prism 3100 Genetic Analyzer (Perkin Elmer). Oligonucleotide primers used in this work are listed in Table S3. Primers annealing to SGI1 were designed according to the published sequence AF261825 (GenBank). The β‐galactosidase assays were performed in 5 parallels (*n* = 5) according to (Miller, 1972) except that the cultures were grown at 37°C to an OD_600_ ~ 0.3 in LB broth and diluted at a ratio of 1:1 with Z buffer.

### 4.2 Mating assays

In the mating assays, *E. coli* strains TG1Nal or Tuner harboring R55 or R55^ΔTn^ *^6187^* helper plasmid were used as a donor with strains TG2 or TG90 recipients. In the complementation assays, the donor strains contained also the KO S021 or wt SGI1‐C integrated into the chromosomal *attB* in *trmE* and one of the p15A‐based complementing plasmids. In the mobilization tests for mutant, wt, or truncated mob_SGI1_ regions, the donor strain carried the appropriate chromosomal SGI1‐C derivative or the test plasmid harboring the mutagenized or truncated mob_SGI1_ fragment. Overnight cultures (1–2 × 10^9^ cells/ml) of donor and recipient strains grown in LB under selection for the chromosomal resistance markers and markers of all additional components (Plasmids, SGI1) present in the donor strains were mixed, centrifuged for 1 min, washed with 0.5 ml 0.9% NaCl, and spread onto LB agar plates, which were incubated for 6 hr at 37°C. For compensation of the lower fitness of the donors (mainly based on the incompatibility of SGI1 and IncC helper plasmid and the genetic load due to the plasmid content), 3:1 donor/recipient ratio was applied in the matings. At the end of incubation, the bacterial lawn was suspended in 4 ml 0.9% NaCl solution, and 5 μl of serial dilutions was dropped onto selective LB plates to determine the titers of donor, recipient, and transconjugant cells. In cases of low transfer frequency (e.g., negative controls), rare transconjugants were detected by spreading 100 μl (instead of dropping 5 μl) of undiluted bacterial suspension obtained from the mating LB plates. In cases where no transconjugants were detected, the detection limit was calculated as 1 transconjugant/ml divided by the donor or the recipient titer and this fraction was regarded as a threshold value of the detectable minimal transfer frequency in that assay. Titers of recipients and donors were determined by selection for their sole chromosomal marker (Tc^R^ and Nal^R^, respectively) except in case of using the *E. coli* strain Tuner (Figure 3b) where, in the absence of chromosomal marker, the donor titers were determined by selection for markers of all the three plasmids of the donor strain (Cm^R^, Km^R^, and Ap^R^). The transconjugants were selected for the chromosomal marker of the recipient and the transferred marker of SGI1‐C or the test plasmid (TcSp or TcKm). The transfer frequencies were calculated as ratios of transconjugant/recipient and transconjugant/donor CFUs from at least four independent parallels (*n* ≥ 4).

### 4.3 Targeted gene KO

All KO mutagenesis were carried out according to the one‐step gene inactivation method (Datsenko & Wanner, 2000). The PCR fragments for KO mutagenesis were amplified from pKD3 template plasmid using the following primers: KOS021 in SGI1‐C and the cloned mob_SGI1_ in pFOL1372—delS021for‐delS021rev; SGI1‐C^Δ2^—deloriTfor‐delS022rev; SGI1‐C^Δ5^—delRNA3for‐deloriTrev; SGI1‐C^Δ6^—delRNA4for‐deloriTrev; and SGI1‐C^Δ7^—deloriTfor‐delS022upstream_rev (Table S3). For promoting the gene replacement, λ Red recombinase was expressed at 30°C for 1.5 hr from pKD46 using 1% L‐arabinose as an inductor. The Cm^R^ cassette was removed from the chromosomal KO alleles by expressing the Flp recombinase from the thermo‐inducible expression plasmid pCP20 at 42°C, or by digestion with XbaI (present in FRT sites) followed by religation in the case of pFOL1372 (resulting in pJKI773). In the KO mutant ORFs S019–S022, 83‐bp sequences near the 5′‐end of each ORF were replaced with an 84‐bp sequence deriving from the PCR template plasmid pKD3 after Flp‐induced deletion of the resistance marker.

For the generation of TG1Nal::SGI1‐C^ΔP5^ strain the mutagenesis PCR fragment was amplified from the template plasmid pMNI18 with primers KO_promS022for–KO_promS022rev. These primers and pMNI18 were designed to ensure that the amplicon contained the Km^R^ gene and the appropriately oriented *rrnB*_T1T2_ terminators. The amplified *rrnB*_T1T2_::Km^R^ cassette was knocked in to SGI1‐C by the one‐step gene inactivation method, replacing the P5 promoter (18,461–18,495 bp). Then, SGI1‐C^ΔP5^ was mobilized by R55^ΔTn^ *^6187^* into TG1Nal::*repA*_SGI1_ host strain through the Tc^R^ TG90 strain in a two‐step mating process.

### 4.4 RNA isolation

For primer extension assays, total RNA was extracted from *E. coli* TG1 harboring pMSZ946 or pJKI990 (as a negative control) using RNeasy Mini Kit (QIAgen) according to the manufacturer's recommendations. For Northern analysis, RNA was isolated from strain TG1Nal::*repA*_SGI1_ (Szabó et al., 2021) lacking SGI1 (‐) or containing ΔP5 mutant or wt SGI1‐C using RNeasy Midi Kit (QIAgen). The latter RNA sample was used for RNA ligation for determining the 5′‐ and 3′‐end of the transcripts.

### 4.5 Primer extension

RevertAid H Minus first‐strand cDNA synthesis kit (Thermo Scientific) was used for the extension, while Sequenase version 2.0 DNA sequencing kit (USB) was applied to generate sequence ladder for the test plasmid pMSZ946. Both kits were used as recommended by the manufacturers. The primers lacZoutE and pUCfor24 used for both reactions were labeled as described by Murányi et al. (2016). Products of extension and sequencing reactions were run on a 6% denaturing polyacrylamide gel at 1,800 V. The gel was exposed to a storage phosphor screen and scanned on Storm 840 PhosphorImager (Amersham Biosciences).

### 4.6 Northern analysis

Five micrograms of total RNA samples were separated on a 5% or 8% denaturing TBE‐polyacrylamide gel containing 8 M urea 200 V (~12 mA). Gels were stained for 20 min in TBE buffer containing 0.5 μg/ml ethidium bromide, photographed next to a gel ruler under UV light, then electro‐transferred (300 mA, 60 min) and crosslinked onto Hybond‐N+ nylon membranes (Amersham) using the LKB 2117 Multiphore electroblotting unit and Amersham ultraviolet crosslinker, respectively. The template DNA for generation of the 3′‐ and 5′‐end hybridization probes were amplified using primer pairs T3_SGI_17741for–T7_SGI_17994rev and T3_SGI_18219for–T7_SGI_18628rev, respectively. The RNA probes were synthesized with T3 polymerase and the blots were hybridized and developed using the DIG Northern Starter Kit (Roche) according to the manufacturer's protocol.

### 4.7 Determination of 5′‐ and 3′‐ends of the RNA transcripts synthesized from promoters upstream of ORF S022

This experiment was based on the method described by Kuhn and Binder (2002). Five microliters of total RNA isolated from strain TG1Nal::*repA*_SGI1_ (Szabó et al., 2021) containing SGI1‐C^WT^ was denatured at 92°C for 2 min, cooled on ice for 1 min, and circularized using 40 units of T4 RNA Ligase (Thermo Fisher), 40 units of RNase OUT (Invitrogen) in 1× T4 Ligase buffer (Thermo Fisher) in a final volume of 25 μl at 37°C for 1 hr. The enzymes were inactivated at 92°C for 10 min and the RNA was precipitated by 96% ethanol and dissolved in 10 μl nuclease‐free water. For reverse transcription, 20 pmole of SGI_S022promseq primer was added to 8 μl of the circularized RNA and annealed at 70°C for 5 min, then the mixture was cooled on ice for 1 min and used as a template for reverse transcription. The reaction was carried out in a final volume of 20 μl using the RevertAid H Minus First‐Strand cDNA Synthesis kit (Fermentas) according to the manufacturer's protocol. For the amplification of the 5′–3′‐end junctions, S022_promfor_Nc and S021promseq primers were used. The reaction mix contained 1 μl of cc. or 10× diluted reaction mix from reverse transcription or, as a negative control, 1 μl of the 4× diluted circularized RNA sample, 1μl dNTP mix (10 mM each), 10 pmol of primers, 1× DreamTaq reaction buffer (ThermoFisher), and 1.5 u DreamTaq polymerase supplemented to 2.5 mM MgCl_2_ in a final volume of 25 μl. The cycling conditions were as follows: initial denaturation at 94°C for 2 min, amplification in 35 cycles at 94°C for 20 s, 55°C for 30 s, 72°C for 40 s, and a final synthesis at 72°C for 7 min. The 250–300 bp range of the PCR product was isolated from the gel, digested with NcoI, and ligated into the NcoI‐HincII‐digested pGEM‐5Zf(+) (Promega). Individual clones were sequenced on ABI Prism 3100 Genetic Analyzer (Perkin–Elmer).

### 4.8 Bioinformatics

Promoter motifs were predicted by BPROM (Solovyev & Salamov, 2011), BDGP (Reese, 2001), and manual search. All homology searches were performed with the NCBI BLAST server. SGI1‐related elements were identified via a nucleotide BLAST search in GenBank using the SGI1 backbone as a query sequence, which was generated as described (Kiss et al., 2019). The sequence alignment was generated using the MultAlin interface (Corpet, 1988). The sgm‐sRNA secondary structure and potential stem‐loop structures were predicted using the mFold server (Zuker, 2003).

## AUTHOR CONTRIBUTIONS

J.K. conceived the project. I.N., J.K., M.S., and A.H. designed and carried out the experiments and analyzed the data. J.K. prepared the figures and wrote the paper and all authors reviewed the manuscript.

## Supporting information

Supplementary Material

Fig S8

## Data Availability

The data that support the findings of this study are available in the supplementary material of this article.

## References

[mmi14846-bib-0001] Ambrose, S.J. , Harmer, C.J. & Hall, R.M. (2018) Evolution and typing of IncC plasmids contributing to antibiotic resistance in Gram‐negative bacteria. Plasmid, 99, 40–55. Available from: http://www.ncbi.nlm.nih.gov/pubmed/30081066 30081066 10.1016/j.plasmid.2018.08.001

[mmi14846-bib-0002] Bandyra, K.J. , Said, N. , Pfeiffer, V. , Górna, M.W. , Vogel, J. & Luisi, B.F. (2012) The seed region of a small RNA drives the controlled destruction of the target mRNA by the endoribonuclease RNase E. Molecular Cell, 47, 943–953. Available from: http://www.ncbi.nlm.nih.gov/pubmed/22902561 22902561 10.1016/j.molcel.2012.07.015 PMC3469820

[mmi14846-bib-0003] Bechhofer, D.H. & Deutscher, M.P. (2019) Bacterial ribonucleases and their roles in RNA metabolism. Critical Reviews in Biochemistry and Molecular Biology, 54, 242–300. Available from: http://www.ncbi.nlm.nih.gov/pubmed/31464530 31464530 10.1080/10409238.2019.1651816 PMC6776250

[mmi14846-bib-0004] Bellanger, X. , Payot, S. , Leblond‐Bourget, N. & Guédon, G. (2014) Conjugative and mobilizable genomic islands in bacteria: evolution and diversity. FEMS Microbiology Reviews, 38, 720–760. Available from: http://www.ncbi.nlm.nih.gov/pubmed/24372381 24372381 10.1111/1574-6976.12058

[mmi14846-bib-0005] Blower, T.R. , Short, F.L. , Rao, F. , Mizuguchi, K. , Pei, X.Y. , Fineran, P.C. et al. (2012) Identification and classification of bacterial Type III toxin‐antitoxin systems encoded in chromosomal and plasmid genomes. Nucleic Acids Research, 40, 6158–6173. Available from: http://www.ncbi.nlm.nih.gov/pubmed/22434880 22434880 10.1093/nar/gks231 PMC3401426

[mmi14846-bib-0006] Bossi, L. & Figueroa‐Bossi, N. (2016) Competing endogenous RNAs: a target‐centric view of small RNA regulation in bacteria. Nature Reviews Microbiology, 14, 775–784. Available from: 10.1038/nrmicro.2016.129 27640758

[mmi14846-bib-0007] Boyd, D. , Cloeckaert, A. , Chaslus‐Dancla, E. & Mulvey, M.R. (2002) Characterization of variant Salmonella genomic island 1 multidrug resistance regions from serovars Typhimurium DT104 and Agona. Antimicrobial Agents and Chemotherapy, 46(6), 1714–1722. Available from: https://journals.asm.org/doi/10.1128/AAC.46.6.1714‐1722.2002 12019080 10.1128/AAC.46.6.1714-1722.2002 PMC127246

[mmi14846-bib-0008] Boyd, D. , Peters, G.A. , Cloeckaert, A. , Boumedine, K.S. , Chaslus‐Dancla, E. , Imberechts, H. et al. (2001) Complete nucleotide sequence of a 43‐kilobase genomic island associated with the multidrug resistance region of Salmonella enterica serovar Typhimurium DT104 and its identification in phage type DT120 and serovar Agona. Journal of Bacteriology, 183, 5725–5732. Available from: http://www.ncbi.nlm.nih.gov/pubmed/11544236 11544236 10.1128/JB.183.19.5725-5732.2001 PMC95465

[mmi14846-bib-0009] Brantl, S. (2012) Bacterial type I toxin‐antitoxin systems. RNA Biology, 9, 1488–1490. Available from: http://www.tandfonline.com/doi/abs/ 10.4161/rna.23045 23324552

[mmi14846-bib-0010] Carr, S.B. , Phillips, S.E.V. & Thomas, C.D. (2016) Structures of replication initiation proteins from staphylococcal antibiotic resistance plasmids reveal protein asymmetry and flexibility are necessary for replication. Nucleic Acids Research, 44(5), 2417–2428. Available from: https://academic.oup.com/nar/article‐lookup/doi/10.1093/nar/gkv1539 26792891 10.1093/nar/gkv1539 PMC4797284

[mmi14846-bib-0011] Carraro, N. , Durand, R. , Rivard, N. , Anquetil, C. , Barrette, C. , Humbert, M. et al. (2017) Salmonella genomic island 1 (SGI1) reshapes the mating apparatus of IncC conjugative plasmids to promote self‐propagation. PLoS Genetics, 13, e1006705. Available from: 10.1371/journal.pgen.1006705 28355215 PMC5389848

[mmi14846-bib-0012] Carraro, N. , Matteau, D. , Luo, P. , Rodrigue, S. & Burrus, V. (2014) The master activator of IncA/C conjugative plasmids stimulates genomic islands and multidrug resistance dissemination. PLOS Genetics, 10, e1004714. Available from: http://www.ncbi.nlm.nih.gov/pubmed/25340549 25340549 10.1371/journal.pgen.1004714 PMC4207636

[mmi14846-bib-0013] Chandler, M. , de la Cruz, F. , Dyda, F. , Hickman, A.B. , Moncalian, G. & Ton‐Hoang, B. (2013) Breaking and joining single‐stranded DNA: the HUH endonuclease superfamily. Nature Reviews Microbiology, 11, 525–538. Available from: 10.1038/nrmicro3067 23832240 PMC6493337

[mmi14846-bib-0014] Corpet, F. (1988) Multiple sequence alignment with hierarchical clustering. Nucleic Acids Research, 16(22), 10881–10890. Available from: 10.1093/nar/16.22.10881 2849754 PMC338945

[mmi14846-bib-0015] Cummins, M.L. , Hamidian, M. & Djordjevic, S.P. (2020) Salmonella genomic island 1 is broadly disseminated within gammaproteobacteriaceae. Microorganisms, 8(2), 1–10. Available from: https://www.mdpi.com/2076‐2607/8/2/161 10.3390/microorganisms8020161 PMC7074787 31979280

[mmi14846-bib-0016] Cummins, M.L. , Roy Chowdhury, P. , Marenda, M.S. , Browning, G.F. & Djordjevic, S.P. (2019) Salmonella Genomic Island 1B variant found in a sequence type 117 avian pathogenic *Escherichia coli* isolate. mSphere, 4(3), e00169‐19. Available from: 10.1128/mSphere.00169-19 31118300 PMC6531882

[mmi14846-bib-0017] de Curraize, C. , Siebor, E. , Neuwirth, C. & Hall, R.M. (2020) SGI0, a relative of Salmonella genomic islands SGI1 and SGI2, lacking a class 1 integron, found in Proteus mirabilis. Plasmid, 107, 102453. Available from: https://linkinghub.elsevier.com/retrieve/pii/S0147619X19301295 31705941 10.1016/j.plasmid.2019.102453

[mmi14846-bib-0018] Datsenko, K.A. & Wanner, B.L. (2000) One‐step inactivation of chromosomal genes in *Escherichia coli* K‐12 using PCR products. Proceedings of the National Academy of Sciences of the United States of America, 97(12), 6640–6645. Available from: http://www.pnas.org/cgi/doi/10.1073/pnas.120163297 10829079 10.1073/pnas.120163297 PMC18686

[mmi14846-bib-0019] Douard, G. , Praud, K. , Cloeckaert, A. & Doublet, B. (2010) The Salmonella genomic island 1 is specifically mobilized in trans by the IncA/C multidrug resistance plasmid family. PLoS One, 5(12), e15312. Available from: https://dx.plos.org/10.1371/journal.pone.0015302 21187963 10.1371/journal.pone.0015302 PMC3004903

[mmi14846-bib-0020] Durand, R. , Deschênes, F. & Burrus, V. (2021a) Genomic islands targeting dusA in Vibrio species are distantly related to Salmonella Genomic Island 1 and mobilizable by IncC conjugative plasmids. PLoS Genetics, 17(8), e1009669. Available from: https://dx.plos.org/10.1371/journal.pgen.1009669 34415925 10.1371/journal.pgen.1009669 PMC8409611

[mmi14846-bib-0021] Durand, R. , Huguet, K.T. , Rivard, N. , Carraro, N. , Rodrigue, S. & Burrus, V. (2021b) Crucial role of Salmonella genomic island 1 master activator in the parasitism of IncC plasmids. Nucleic Acids Research, 49(14), 7807–7824. Available from: https://academic.oup.com/nar/article/49/14/7807/6212760 33834206 10.1093/nar/gkab204 PMC8373056

[mmi14846-bib-0022] Fineran, P.C. , Blower, T.R. , Foulds, I.J. , Humphreys, D.P. , Lilley, K.S. & Salmond, G.P.C. (2009) The phage abortive infection system, ToxIN, functions as a protein‐RNA toxin‐antitoxin pair. Proceedings of the National Academy of Sciences of the United States of America, 106, 894–899. Available from: http://www.ncbi.nlm.nih.gov/pubmed/19124776 19124776 10.1073/pnas.0808832106 PMC2630095

[mmi14846-bib-0023] Finlay, B.B. , Frost, L.S. & Paranchych, W. (1986) Nucleotide sequences of the R1–19 plasmid transfer genes traM, finP, traJ, and traY and the traYZ promoter. Journal of Bacteriology, 166(2), 368–374. Available from: https://journals.asm.org/doi/10.1128/jb.166.2.368‐374.1986 3009392 10.1128/jb.166.2.368-374.1986 PMC214613

[mmi14846-bib-0024] Fröhlich, K.S. , Papenfort, K. , Fekete, A. & Vogel, J. (2013) A small RNA activates CFA synthase by isoform‐specific mRNA stabilization. EMBO Journal, 32, 2963–2979. Available from: http://www.ncbi.nlm.nih.gov/pubmed/24141880 24141880 10.1038/emboj.2013.222 PMC3831309

[mmi14846-bib-0025] Garcillán‐Barcia, M.P. , Francia, M.V. & De La Cruz, F. (2009) The diversity of conjugative relaxases and its application in plasmid classification. FEMS Microbiology Reviews, 33(3), 657–687. Available from: https://academic.oup.com/femsre/article‐lookup/doi/10.1111/j.1574‐6976.2009.00168.x 19396961 10.1111/j.1574-6976.2009.00168.x

[mmi14846-bib-0027] Golding, G.R. , Olson, A.B. , Doublet, B. , Cloeckaert, A. , Christianson, S. , Graham, M.R. et al. (2007) The effect of the Salmonella genomic island 1 on in vitro global gene expression in Salmonella enterica serovar Typhimurium LT2. Microbes and Infection, 9, 21–27. Available from: http://www.ncbi.nlm.nih.gov/pubmed/17194608 17194608 10.1016/j.micinf.2006.10.004

[mmi14846-bib-0028] Guzmán‐Herrador, D.L. & Llosa, M. (2019) The secret life of conjugative relaxases. Plasmid, 104, 102415. Available from: 10.1016/j.plasmid.2019.102415 31103521

[mmi14846-bib-0029] Harmer, C.J. & Hall, R.M. (2015) The A to Z of A/C plasmids. Plasmid, 80, 63–82. Available from: 10.1016/j.plasmid.2015.04.003 25910948

[mmi14846-bib-0030] Harmer, C.J. , Hamidian, M. , Ambrose, S.J. & Hall, R.M. (2016) Destabilization of IncA and IncC plasmids by SGI1 and SGI2 type Salmonella genomic islands. Plasmid, 87–88, 51–57. Available from: 10.1016/j.plasmid.2016.09.003 27620651

[mmi14846-bib-0031] Hegyi, A. , Szabó, M. , Olasz, F. & Kiss, J. (2017) Identification of oriT and a recombination hot spot in the IncA/C plasmid backbone. Scientific Reports, 7, 10595. Available from: http://www.nature.com/articles/s41598‐017‐11097‐0 28878309 10.1038/s41598-017-11097-0 PMC5587640

[mmi14846-bib-0032] Heilers, J.H. , Reiners, J. , Heller, E.M. , Golzer, A. , Smits, S.H.J. , Does, C.V. et al. (2019) DNA processing by the MOBH family relaxase TraI encoded within the gonococcal genetic island. Nucleic Acids Research, 47(15), 8136–8153. Available from: https://academic.oup.com/nar/article/47/15/8136/5528732 31276596 10.1093/nar/gkz577 PMC6736028

[mmi14846-bib-0033] Holmqvist, E. , Berggren, S. & Rizvanovic, A. (2020) RNA‐binding activity and regulatory functions of the emerging sRNA‐binding protein ProQ. Biochimica et Biophysica Acta (BBA) ‐ Gene Regulatory Mechanisms, 1863(9), 194596. Available from: http://www.ncbi.nlm.nih.gov/pubmed/32565402 32565402 10.1016/j.bbagrm.2020.194596

[mmi14846-bib-0034] Huguet, K.T. , Gonnet, M. , Doublet, B. & Cloeckaert, A. (2016) A toxin antitoxin system promotes the maintenance of the IncA/C‐mobilizable Salmonella Genomic Island 1. Scientific Reports, 6(1), 32285. Available from: http://www.nature.com/articles/srep32285 27576575 10.1038/srep32285 PMC5006074

[mmi14846-bib-0035] Huguet, K.T. , Rivard, N. , Garneau, D. , Palanee, J. & Burrus, V. (2020) Replication of the Salmonella Genomic Island 1 (SGI1) triggered by helper IncC conjugative plasmids promotes incompatibility and plasmid loss. PLoS Genetics, 16, e1008965. Available from: https://dx.plos.org/10.1371/journal.pgen.1008965 32760058 10.1371/journal.pgen.1008965 PMC7433901

[mmi14846-bib-0036] Jørgensen, M.G. , Pettersen, J.S. & Kallipolitis, B.H. (2020) sRNA‐mediated control in bacteria: an increasing diversity of regulatory mechanisms. Biochimica et Biophysica Acta (BBA) ‐ Gene Regulatory Mechanisms, 1863(5), 194504. Available from: 10.1016/j.bbagrm.2020.194504 32061884

[mmi14846-bib-0037] Kiss, J. , Nagy, B. & Olasz, F. (2012) Stability, entrapment and variant formation of Salmonella genomic island 1. PLoS One, 7, e32497. Available from: http://dx.plos.org/10.1371/journal.pone.0032497 22384263 10.1371/journal.pone.0032497 PMC3285670

[mmi14846-bib-0038] Kiss, J. , Papp, P.P. , Szabó, M. , Farkas, T. , Murányi, G. , Szakállas, E. et al. (2015) The master regulator of IncA/C plasmids is recognized by the Salmonella Genomic island SGI1 as a signal for excision and conjugal transfer. Nucleic Acids Research, 43(18), 8735–8745. Available from: https://academic.oup.com/nar/article‐lookup/doi/10.1093/nar/gkv758 26209134 10.1093/nar/gkv758 PMC4605294

[mmi14846-bib-0039] Kiss, J. , Szabó, M. , Hegyi, A. , Douard, G. , Praud, K. , Nagy, I. et al. (2019) Identification and characterization of oriT and two mobilization genes required for conjugative transfer of Salmonella Genomic Island 1. Frontiers in Microbiology, 10, 1–16. Available from: https://www.frontiersin.org/article/10.3389/fmicb.2019.00457/full 30894848 10.3389/fmicb.2019.00457 PMC6414798

[mmi14846-bib-0040] Koraimann, G. , Teferle, K. , Markolin, G. , Woger, W. & Högenauer, G. (1996) The FinOP repressor system of plasmid R1: analysis of the antisense RNA control of traJ expression and conjugative DNA transfer. Molecular Microbiology, 21, 811–821. Available from: http://www.ncbi.nlm.nih.gov/pubmed/8878043 8878043 10.1046/j.1365-2958.1996.361401.x

[mmi14846-bib-0041] Kuhn, J. & Binder, S. (2002) RT‐PCR analysis of 5′ to 3′‐end‐ligated mRNAs identifies the extremities of cox2 transcripts in pea mitochondria. Nucleic Acids Research, 30(2), 439–446. Available from: https://academic.oup.com/nar/article‐lookup/doi/10.1093/nar/30.2.439 11788705 10.1093/nar/30.2.439 PMC99824

[mmi14846-bib-0042] Levings, R.S. , Djordjevic, S.P. & Hall, R.M. (2008) SGI2, a relative of Salmonella genomic island SGI1 with an independent origin. Antimicrobial Agents and Chemotherapy, 52(7), 2529–2537. Available from: https://journals.asm.org/doi/10.1128/AAC.00189‐08 18443113 10.1128/AAC.00189-08 PMC2443934

[mmi14846-bib-0043] Llosa, M. & Alkorta, I. (2017 ) Coupling proteins in type IV secretion. In: Current topics in microbiology and immunology, pp. 143–168. Available from: http://link.springer.com/ 10.1007/978-3-319-75241-9_6 29536358

[mmi14846-bib-0044] Llosa, M. , Gomis‐Rüth, F.X. , Coll, M ., & de la Cruz, F. (2002) Bacterial conjugation: a two‐step mechanism for DNA transport. Molecular Microbiology, 45, 1–8. Available from: http://www.ncbi.nlm.nih.gov/pubmed/12100543 12100543 10.1046/j.1365-2958.2002.03014.x

[mmi14846-bib-0045] Miller, J.H. (1972) Experiment 48 assay of beta‐galactosidase. In: Experiments in molecular genetics. Cold Spring Harbor, NY: Cold Spring Harbor Lab Press. pp. 352–355.

[mmi14846-bib-0046] Murányi, G. , Szabó, M. , Olasz, F. & Kiss, J. (2016) Determination and analysis of the putative AcaCD‐responsive promoters of Salmonella Genomic Island 1. PLoS One, 11, e0164561. Available from: http://dx.plos.org/ 10.1371/journal.pone.0164561 27727307 PMC5058578

[mmi14846-bib-0047] Nowak, K.P. , Sobolewska‐Ruta, A. , Jagiełło, A. , Bierczyńska‐Krzysik, A. , Kierył, P. & Wawrzyniak, P. (2021) Molecular and functional characterization of MobK protein—a novel‐type relaxase involved in mobilization for conjugational transfer of *Klebsiella pneumoniae* plasmid pIGRK. International Journal of Molecular Sciences, 22, 5152. Available from: https://www.mdpi.com/1422‐0067/22/10/5152 34068033 10.3390/ijms22105152 PMC8152469

[mmi14846-bib-0048] Núñez, B. & De La Cruz, F. (2001) Two atypical mobilization proteins are involved in plasmid CloDF13 relaxation. Molecular Microbiology, 39, 1088–1099. Available from: http://www.ncbi.nlm.nih.gov/pubmed/11251827 11251827 10.1046/j.1365-2958.2001.02308.x

[mmi14846-bib-0049] Papenfort, K. , Espinosa, E. , Casadesús, J. & Vogel, J. (2015) Small RNA‐based feedforward loop with AND‐gate logic regulates extrachromosomal DNA transfer in Salmonella. Proceedings of the National Academy of Sciences of the United States of America, 112, E4772–E4781. Available from: http://www.pnas.org/lookup/doi/ 10.1073/pnas.1507825112 26307765 PMC4553797

[mmi14846-bib-0050] Quendera, A.P. , Seixas, A.F. , dos Santos, R.F. , Santos, I. , Silva, J.P.N. , Arraiano, C.M. et al. (2020) RNA‐binding proteins driving the regulatory activity of small non‐coding RNAs in bacteria. Frontiers in Molecular Biosciences, 7, 1–9. Available from: https://www.frontiersin.org/article/ 10.3389/fmolb.2020.00078/full 32478092 PMC7237705

[mmi14846-bib-0051] Reese, M.G. (2001) Application of a time‐delay neural network to promoter annotation in the *Drosophila melanogaster* genome. Computers & Chemistry, 26, 51–56. Available from: http://www.ncbi.nlm.nih.gov/pubmed/11765852 11765852 10.1016/s0097-8485(01)00099-7

[mmi14846-bib-0052] Richards, J. & Belasco, J.G. (2021) Riboswitch control of bacterial RNA stability. Molecular Microbiology, 116(2), 361–365. Available from: https://onlinelibrary.wiley.com/doi/10.1111/mmi.14723 33797153 10.1111/mmi.14723 PMC10367942

[mmi14846-bib-0053] Sambrook, J. , Fritsch, E.F. & Maniatis, T. (1989) Molecular cloning: a laboratory manual. Cold Spring Harbor Laboratory Press.

[mmi14846-bib-0054] Schultz, E. , Barraud, O. , Madec, J.‐Y. , Haenni, M. , Cloeckaert, A. , Ploy, M.‐C. et al. (2017) Multidrug resistance Salmonella Genomic Island 1 in a *Morganella* *morganii* Subsp. morganii human clinical isolate from France. mSphere, 2, e00118‐17. Available from: https://journals.asm.org/doi/10.1128/mSphere.00118‐17 28435889 10.1128/mSphere.00118-17 PMC5397566

[mmi14846-bib-0055] Shokeen, S. , Johnson, C.M. , Greenfield, T.J. , Manias, D.A. , Dunny, G.M. & Weaver, K.E. (2010) Structural analysis of the Anti‐Q‐Qs interaction: RNA‐mediated regulation of *E. faecalis* plasmid pCF10 conjugation. Plasmid, 64(1), 26–35. Available from: https://linkinghub.elsevier.com/retrieve/pii/S0147619X10000259 20332003 10.1016/j.plasmid.2010.03.002 PMC2892192

[mmi14846-bib-0057] Siebor, E. , de Curraize, C. , Amoureux, L. & Neuwirth, C. (2016) Mobilization of the Salmonella genomic island SGI1 and the Proteus genomic island PGI1 by the A/C2 plasmid carrying blaTEM‐24 harboured by various clinical species of Enterobacteriaceae. Journal of Antimicrobial Chemotherapy, 71(8), 2167–2170. Available from: https://academic.oup.com/jac/article‐lookup/doi/10.1093/jac/dkw151 27150396 10.1093/jac/dkw151

[mmi14846-bib-0058] Siebor, E. , de Curraize, C. & Neuwirth, C. (2019) Identification of AGI1‐A, a variant of Acinetobacter genomic island 1 (AGI1), in a French clinical isolate belonging to the Enterobacter cloacae complex. Journal of Antimicrobial Chemotherapy, 74(2), 311–314. Available from: https://academic.oup.com/jac/article/74/2/311/5166736 30412264 10.1093/jac/dky442

[mmi14846-bib-0059] Simon, R. , Priefer, U. & Pühler, A. (1983) A broad host range mobilization system for in vivo genetic engineering: transposon mutagenesis in gram negative bacteria. Bio/Technology, 1, 784–791. Available from: http://www.nature.com/doifinder/ 10.1038/nbt1183-784

[mmi14846-bib-0060] Soliman, A.M. , Ramadan, H. , Ghazy, E. , Yu, L. , Hisatsune, J. , Kayama, S. et al. (2020) Emergence of Salmonella Genomic Island 1 variant SGI1‐C in a multidrug‐resistant clinical isolate of *Klebsiella pneumoniae* ST485 from Egypt. Antimicrobial Agents and Chemotherapy, 64, 1–4. Available from: http://www.ncbi.nlm.nih.gov/pubmed/32660995 10.1128/AAC.01055-20 PMC7449224 32660995

[mmi14846-bib-0061] Soliman, A.M. , Shimamoto, T. , Nariya, H. & Shimamoto, T. (2018) Emergence of Salmonella Genomic Island 1 Variant SGI1‐W in a clinical isolate of *Providencia stuartii* from Egypt. Antimicrobial Agents and Chemotherapy, 63(1), e01793‐18. Available from: https://journals.asm.org/doi/epub/10.1128/AAC.01793‐18 30348665 10.1128/AAC.01793-18 PMC6325238

[mmi14846-bib-0062] Solovyev, V. & Salamov, A. (2011) Automatic annotation of microbial genomes and metagenomic sequences. In: Li, R.W. (Ed.) Metagenomics and its applications in agriculture, biomedicine and environmental studies. Nova Science Publishers. pp. 61–78. Available from: https://www.researchgate.net/publication/259450599_V_Solovyev_A_Salamov_2011_Automatic_Annotation_of_Microbial_Genomes_and_Metagenomic_Sequences_In_Metagenomics_and_its_Applications_in_Agriculture_Biomedicine_and_Environmental_Studies_Ed_RW_Li_Nova_Sc

[mmi14846-bib-0063] Szabó, M. , Murányi, G. & Kiss, J. (2021) IncC helper dependent plasmid‐like replication of Salmonella Genomic Island 1. Nucleic Acids Research, 49(2), 832–846. Available from: https://academic.oup.com/nar/article/49/2/832/6066640 33406256 10.1093/nar/gkaa1257 PMC7826253

[mmi14846-bib-0064] Wagner, E.G.H. , Altuvia, S. & Romby, P. (2002) Antisense RNAs in bacteria and their genetic elements. Advances in Genetics, 46, 361–398. Available from: http://www.ncbi.nlm.nih.gov/pubmed/11931231 11931231 10.1016/s0065-2660(02)46013-0

[mmi14846-bib-0065] Wagner, E.G.H. & Romby, P. (2015) Small RNAs in bacteria and archaea: who they are, what they do, and how they do it. Advances in Genetics, 90, 133–208. Available from: 10.1016/bs.adgen.2015.05.001 26296935

[mmi14846-bib-0066] Wagner, E.G.H. & Simons, R.W. (1994) Antisense RNA control in bacteria, phages, and plasmids. Annual Review of Microbiology, 48, 713–742. Available from: http://www.annualreviews.org/doi/ 10.1146/annurev.mi.48.100194.003433 7826024

[mmi14846-bib-0067] Waters, J.L. & Salyers, A.A. (2012) The small RNA RteR inhibits transfer of the Bacteroides conjugative transposon CTnDOT. Journal of Bacteriology, 194, 5228–5236. Available from: http://www.ncbi.nlm.nih.gov/pubmed/22821972 22821972 10.1128/JB.00941-12 PMC3457204

[mmi14846-bib-0068] Wisniewski, J.A. , Traore, D.A. , Bannam, T.L. , Lyras, D. , Whisstock, J.C. & Rood, J.I. (2016) TcpM: a novel relaxase that mediates transfer of large conjugative plasmids from *Clostridium perfringens* . Molecular Microbiology, 99(5), 884–896. Available from: https://onlinelibrary.wiley.com/doi/10.1111/mmi.13270 26560080 10.1111/mmi.13270

[mmi14846-bib-0069] Wu, W. , Feng, Y. , Tang, G. , Qiao, F. , McNally, A. & Zong, Z. (2019) NDM Metallo‐β‐lactamases and their bacterial producers in health care settings. Clinical Microbiology Reviews, 32, e00115‐18. Available from: http://www.ncbi.nlm.nih.gov/pubmed/30700432 30700432 10.1128/CMR.00115-18 PMC6431124

[mmi14846-bib-0070] Zhang, X. , Wang, C. , Feng, Y. , Long, H. & Zong, Z. (2020) A pandrug‐resistant providencia carrying two blaIMP carbapenemase‐encoding genes including blaIMP‐69, a New blaIMP variant, on a newly identified worldwide‐distributed INCC plasmid. Journal of Infectious Diseases, 221, S253–S256. Available from: https://academic.oup.com/jid/article/221/Supplement_2/S253/5807661 32176782 10.1093/infdis/jiz476

[mmi14846-bib-0071] Zheng, Z. , Cheng, Q. , Chan, E.W. & Chen, S. (2020) Genetic and biochemical characterization of VMB‐1, a novel metallo‐β‐lactamase encoded by a conjugative, broad‐host range IncC plasmid from Vibrio spp. Advanced Biosystems, 4, 1900221. Available from: https://onlinelibrary.wiley.com/doi/abs/ 10.1002/adbi.201900221 32293144

[mmi14846-bib-0072] Zuker, M. (2003) Mfold web server for nucleic acid folding and hybridization prediction. Nucleic Acids Research, 31(13), 3406–3415. Available from: https://academic.oup.com/nar/article‐lookup/doi/10.1093/nar/gkg595 12824337 10.1093/nar/gkg595 PMC169194

