# Supplementary material for: Salmonella Genomic Island 1 requires a self‐encoded small RNA for mobilization
Source: Mol Microbiol. 2021 Nov 25;116(6):1533–51. doi: 10.1111/mmi.14846 (PMC9299015; doi:10.1111/mmi.14846)
Supplement: Supplementary file 1 — Supplementary Material [file MMI-116-1533-s002.pdf]

**Supplementary information**

# *Salmonella* Genomic Island 1 requires a self-encoded small RNA for mobilization

István Nagy, Mónika Szabó, Anna Hegyi, János Kiss

Content:

Supplementary Figure S1
Supplementary Figure S2
Supplementary Figure S3
Supplementary Figure S4
Supplementary Figure S5
Supplementary Figure S6
Supplementary Figure S7

Supplementary Table S1
Supplementary Table S2
Supplementary Table S3

Supplementary Methods

The large image of Supplementary Figure S8 is available as a separate file.

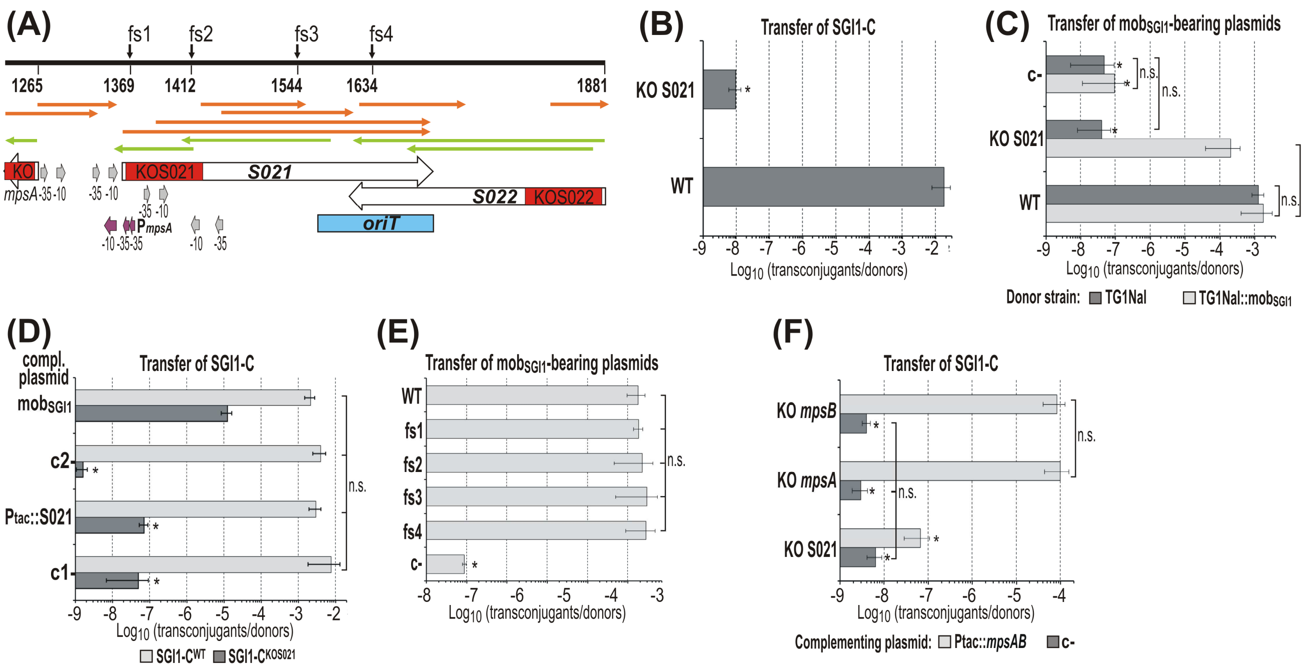


**Figure S1.** Transfer frequencies expressed as the transconjugant per donor CFUs for the mating assays presented on Fig. 2. The donors were selected according to their sole chromosomal marker $Nal^R$. Paired t-test was used to calculate the significance of the differences, n.s. not significant ($P \geq 0.05$). Asterisks, unless otherwise specified, indicate that the transconjugant frequency was below the detection limit, transconjugants were not observed. (B) Transfer frequency of SGI1-$C^{KOS021}$ mutant compared to the SGI1-$C^{WT}$. (C) Transfer of test plasmids carrying wt or S021 KO mutant $mob_{SGI1}$ region from TG1Nal and the complementing strain TG1Nal::$mob_{SGI1}$. Asterisks indicate the basal level of plasmid transfer. (D) *Trans*-complementation of SGI1-$C^{KOS021}$ by the expressed S021 protein. The relatively high detection limit was due to the lower donor titers obtained for strains TG1Nal::SGI1-$C^{KOS021}$/R55$^{\Delta Tn\mathit{6187}}$ harbouring the complementing expression plasmids ($P_{tac}$::S021 and c1). (E) Transfer frequencies of the frameshift mutant $mob_{SGI1}$-bearing test plasmids. (F) *Trans*-complementation of SGI1-$C^{KOS021}$ by expression of MpsA and MpsB proteins.

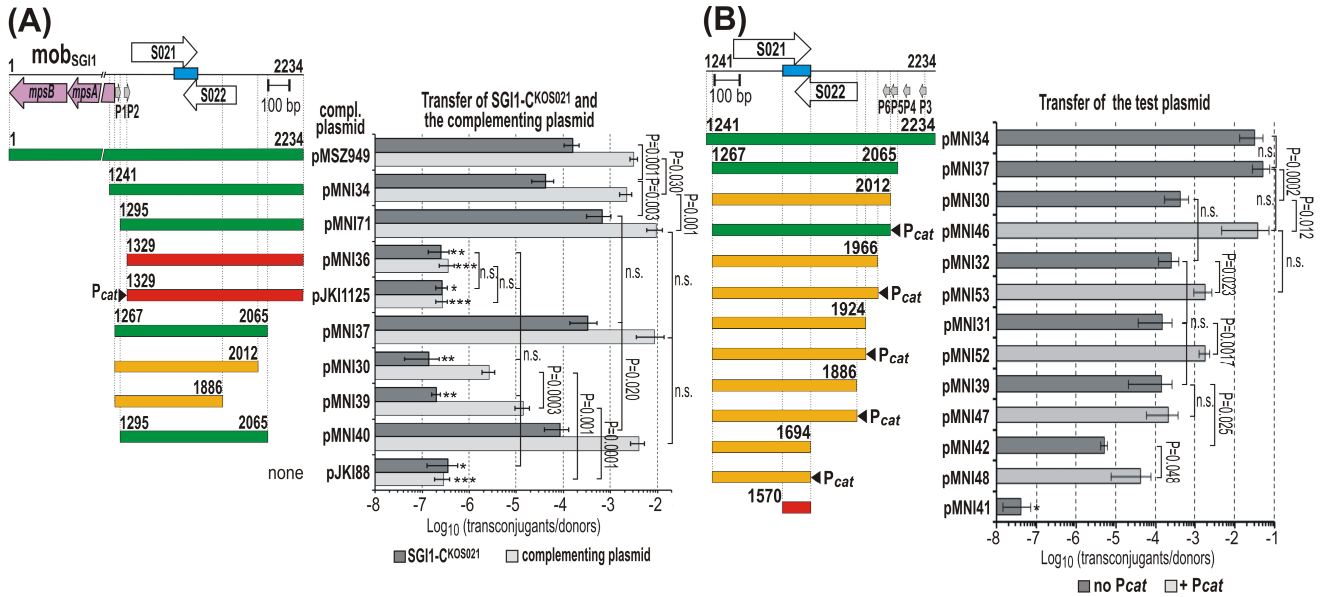


**Figure S2.** Transfer frequencies expressed as the transconjugant per donor CFUs for the mating assays presented on Fig. 3. (A) Localization of the minimal SGI1 region that can complement the SGI1-C$^{KOS021}$ mutant. The donors were selected according to their sole chromosomal marker Nal$^{R}$. * the transconjugant frequency was below the detection limit,no transconjugants were observed. ** several SGI1-C$^{KOS021}$ transconjugant colonies were obtained with a frequency around the detection limit (~5×10$^{-7}$ transconjugant/donor). *** several plasmid transconjugants were obtained with a frequency around the detection limit (~5×10$^{-7}$ transconjugant/donor). The relatively high detection limit was due to the lower donor titers. (B) Promoter mapping and deletion analysis in the 3' end of mob$_{SGI1}$. The titer of the donor *E. coli* Tuner/R55$^{\Delta Tn6187}$ (Sul$^R$Cm$^R$Flo$^R$) containing the MpsAB-producer pJKI879 (Ap$^R$) and one of the test plasmids (Km$^R$) was determined on CmApKm plates in the absence of chromosomal marker. * transconjugant frequency was below the detection limit, no transconjugants were observed.

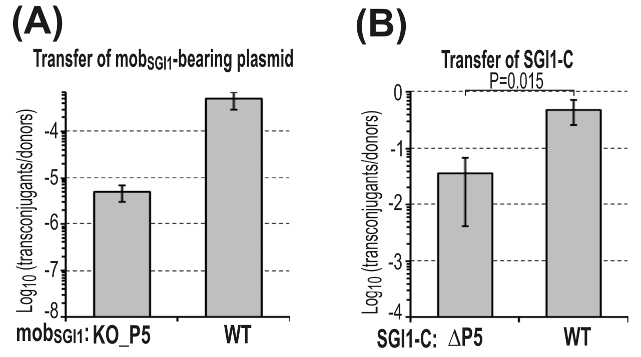


**Figure S3.** Transfer frequencies expressed as the transconjugant per donor CFUs for the mating assays presented on Fig. 4DE. The donors were selected according to their sole chromosomal marker Nal$^R$. (A) Transfer frequency of the mob$_{SGI1}$-bearing plasmid pMNI11, in which the P5 promoter was knocked out (KO_P5). (B) Transfer frequency of SGI1-C$^{\Delta P5}$.

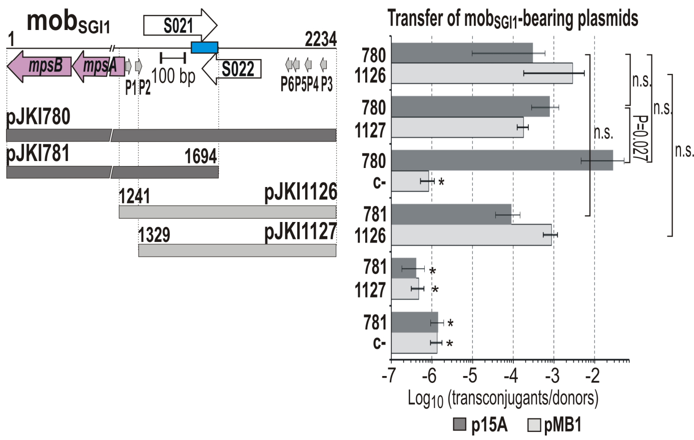


**Figure S4.** Transfer frequencies expressed as the transconjugant per donor CFUs for the mating assay presented on Fig. 5B. Trans-complementation of RNA transcripts synthesized from mob$_{SGI1}$ fragments. Symbols are as in Figs 2 and 3. The titer of donor strains TG1Nal/R55$^{\Delta Tn6187}$ (Nal$^R$Sul$^R$Cm$^R$Flo$^R$) carrying different pairs of the p15A (Sp$^R$) and pMB1 (Km$^R$Ap$^R$) complementing plasmids was determined according to their sole chromosomal marker (Nal$^R$). Transconjugants were selected on TcSp and TcKm plates, respectively. * transfer frequency was below the detection limit, regular transconjugants were not observed.

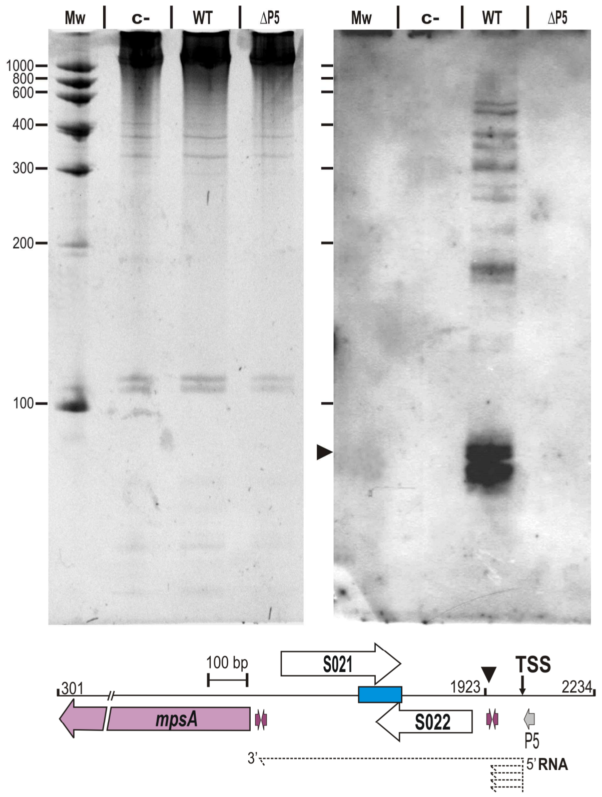


**Figure S5**. Early termination of the primary transcript of sgm-sRNA. Total RNA was extracted from TG1Nal::*repA*$_{SGI1}$ strain lacking SGI1 (-) or containing wt or ΔP5 mutant SGI1-C. The RNA samples were run on 8% denaturing polyacrylamide gel and hybridized with the 5'-probe. The arrowhead points to the abundant 70-80 base long RNA products. The schematic map below the images shows the mob$_{SGI1}$ region where the detected transcripts derive from. The terminator-like IR motifs are indicated by purple arrows, coordinates are as defined for mob$_{SGI1}$ region in Fig. 1, other symbols are as in Fig. 3.

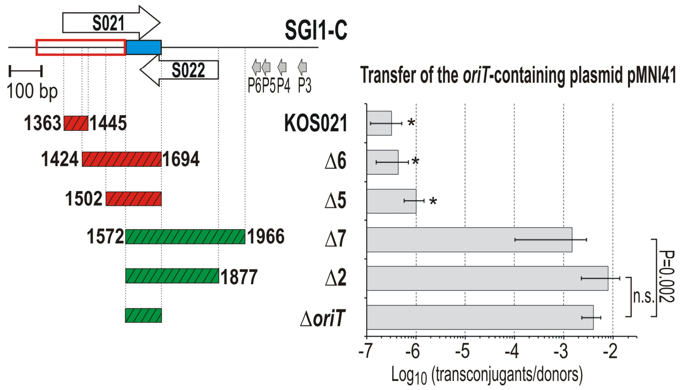


**Figure S6.** Transfer frequencies expressed as the transconjugant per donor CFUs for the mating assay presented on Fig. 5. Mapping of the functional part of the SGI1-encoded sRNA. The donors were selected according to their sole chromosomal marker Nal$^R$, while pMNI41 transconjugants were selected on TcKm plates. Asterisks indicate that transfer frequency of pMNI41 was below the detection limit, transconjugants were not observed.

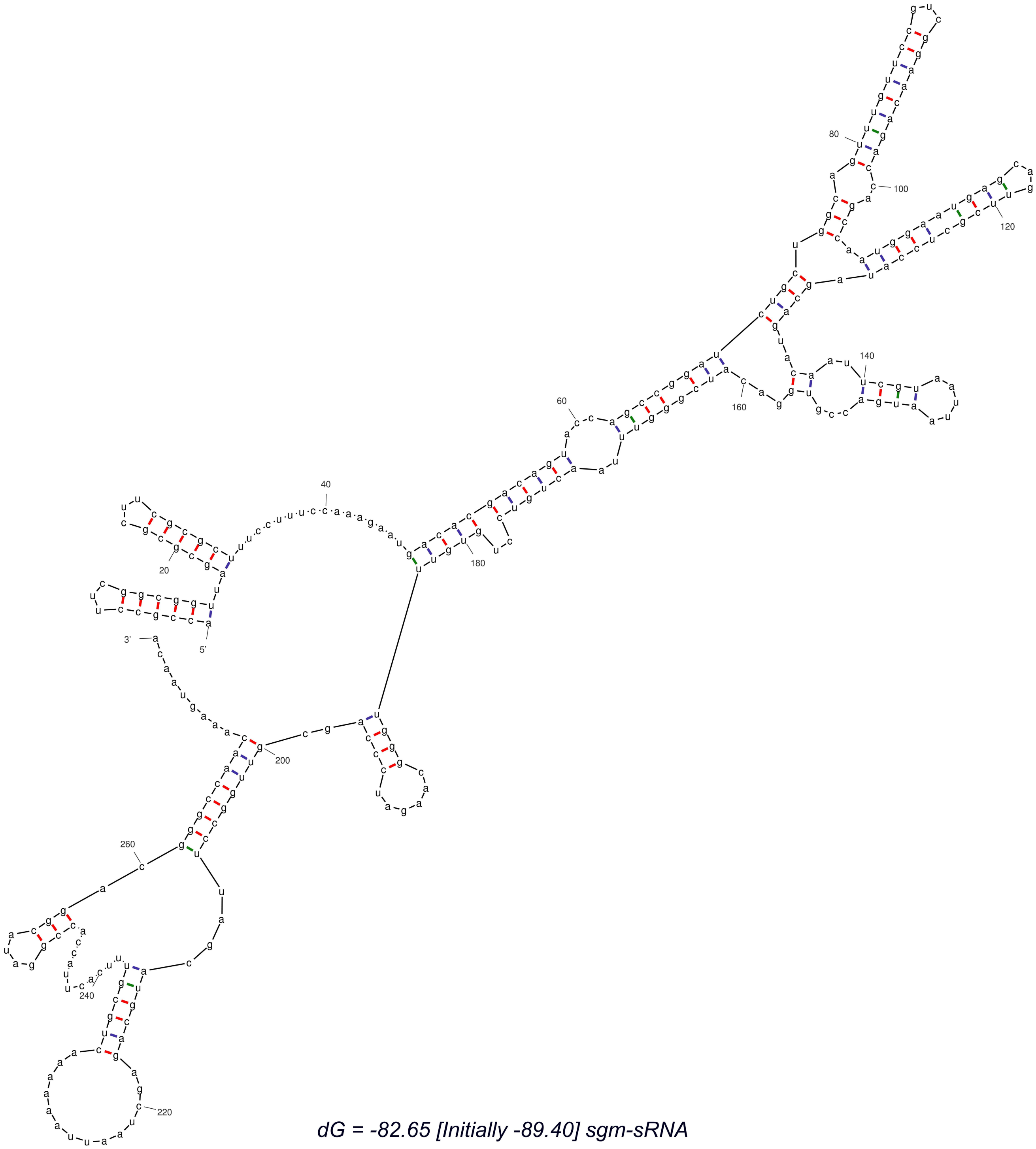


**Figure S7.** Secondary structure prediction for the core domain of sgm-sRNA generated by mFold Web Server. The RNA sequence represents the 1295-1571 bp tract of $mob_{SGI1}$.

**Table S1.** Bacterial strains used in this study.

| *E. coli* strains | Genotype or relevant features[a] | Reference |
|---|---|---|
| TG1 | *supE hsd*Δ5 *thi* Δ(*lac-proAB*) F'[*traD36 proAB*+ *lacIq lacZ*ΔM15] | (Gibson, 1984) |
| TG1Nal | Nal$^R$ derivative of TG1 | (Kiss *et al.*, 2012) |
| TG2 | *supE hsd*Δ5 *thi* Δ(*lac-proAB*)Δ(*srl-recA*)306::Tn*10*(Tc$^R$) F'[*traD36 proAB*+ *lacIq lacZ*ΔM15] | (Sambrook *et al.*, 1989) |
| TG90 | *pcn B80 zad*::Tn*10* (Tc$^R$) derivative of TG1 | (Gonzy-Treboul *et al.*, 1992) |
| TG1Nal/R55 | TG1Nal strain containing R55, Nal$^R$, Ap$^R$, Cm$^R$, Flo$^R$, Su$^R$, Km$^R$, Gm$^R$ | (Kiss *et al.*, 2015) |
| TG1Nal::SGI1-C$^{WT}$ | TG1Nal strain containing SGI1-C variant integrated into *E. coli thdF*, Nal$^R$, Sm$^R$, Sp$^R$, Sul$^R$ | (Kiss *et al.*, 2015) |
| TG1Nal::SGI1-C$^{KOS019}$ | TG1Nal strain containing the *mpsB* (S019) KO mutant SGI1-C, in which the 16656-16739 bp region was replaced by the 84-bp sequence deriving from pKD3 after removing the Cm$^R$ cassette, Nal$^R$, Sm$^R$, Sp$^R$, Sul$^R$ | (Kiss *et al.*, 2019) |
| TG1Nal::SGI1-C$^{KOS020}$ | TG1Nal strain containing the *mpsA* (S020) KO mutant SGI1-C, in which the 17571-17709 bp region was replaced by the 84-bp sequence deriving from pKD3 after removing the Cm$^R$ cassette, Nal$^R$, Sm$^R$, Sp$^R$, Sul$^R$ | (Kiss *et al.*, 2019) |
| TG1Nal::SGI1-C$^{KOS021}$ | TG1Nal strain containing the S021 KO mutant SGI1-C, in which the 17809-17891 bp region was replaced by the 84-bp sequence deriving from pKD3 after removing the Cm$^R$ cassette, Nal$^R$, Sm$^R$, Sp$^R$, Sul$^R$ | this work |
| TG1Nal::SGI1-C$^{\Delta P5}$ | TG1Nal strain containing the ΔP5 mutant SGI1-C, in which the P5 promoter (18461-18495 bp) was replaced by the *rrnB*T1T2::Km$^R$ cassette from pMNI18. Nal$^R$, Sm$^R$, Sp$^R$, Sul$^R$, Km$^R$ | this work |
| TG1Nal::SGI1-C$^{\Delta oriT}$ | TG1Nal strain containing the Δ*oriT* mutant SGI1-C, in which the 18018-18140 bp region was replaced by the the 84-bp sequence deriving from pKD3 after removing the Cm$^R$ cassette, Nal$^R$, Sm$^R$, Sp$^R$, Sul$^R$ | (Kiss *et al.*, 2019) |
| TG1Nal::SGI1-C$^{\Delta 2}$ | TG1Nal strain containing the Δ2 deletion mutant SGI1-C, in which the 18018-18323 bp segment was replaced by the 84-bp sequence deriving from pKD3 after removing the Cm$^R$ cassette, Nal$^R$, Sm$^R$, Sp$^R$, Sul$^R$ | this work |
| TG1Nal::SGI1-C$^{\Delta 5}$ | TG1Nal strain containing the Δ5 deletion mutant SGI1-C, in which the 17948-18140 bp segment was replaced by the the 84-bp sequence deriving from pKD3 after removing the Cm$^R$ cassette, Nal$^R$, Sm$^R$, Sp$^R$, Sul$^R$ | this work |
| TG1Nal::SGI1-C$^{\Delta 6}$ | TG1Nal strain containing the Δ6 deletion mutant SGI1-C, in which the 17870-18140 bp segment was replaced by the the 84-bp sequence deriving from pKD3 after removing the Cm$^R$ cassette, Nal$^R$, Sm$^R$, Sp$^R$, Sul$^R$ | this work |
| TG1Nal::SGI1-C$^{\Delta 7}$ | TG1Nal strain containing the Δ7 deletion mutant SGI1-C, in which the 18018-18412 bp segment was replaced by the the 84-bp sequence deriving from pKD3 after removing the Cm$^R$ cassette, Nal$^R$, Sm$^R$, Sp$^R$, Sul$^R$ | this work |
| TG1Nal::mob$_{SGI1}$/R55 | TG1Nal::[ miniTn*10*::mob$_{SGI1}$-Km$^R$] mobilization helper strain containing R55 and the 16447-18680 bp SGI1 region integrated into the chromosome by miniTn*10* transposition, Nal$^R$, Km$^R$, Ap$^R$, Cm$^R$, Flo$^R$, Sul$^R$, Gm$^R$ | (Kiss *et al.*, 2019) |
| TG1Nal::*repA*$_{SGI1}$ | TG1Nal::[ miniTn*10*::*repA*$_{SGI1}$-Km$^R$] containing the 2162-3115 bp SGI1 region (ORF S003) under the control of P$_{tac}$ promoter and *rrnB* terminator, Nal$^R$,Km$^R$ | (Szabó *et al.*, 2021) |
| TG1Nal::*repA*$_{SGI1}$/SGI1-C$^{WT}$ | TG1Nal::*repA*$_{SGI1}$ strain containing SGI1-C$^{WT}$ as a low copy plasmid, Nal$^R$, Km$^R$, Sm$^R$, Sp$^R$, Sul$^R$ | this work |
| TG1Nal::*repA*$_{SGI1}$/SGI1-C$^{\Delta P5}$ | TG1Nal::*repA*$_{SGI1}$ strain containing SGI1-C$^{\Delta P5}$ as a low copy plasmid, Nal$^R$, Km$^R$, Sm$^R$, Sp$^R$, Sul$^R$ | this work |
| Tuner (DE3)/R55$^{\Delta Tn6187}$ | Derivative of Tuner (DE3) [F$^-$ *ompT hsdS*$_B$(r$_B^-$ m$_B^-$) *gal dcm lacY*1 (DE3)] (Novagen) carrying the R55$^{\Delta Tn6187}$ helper plasmid. | this work |
| S17-1 λpir | S17-1 λ*pir*, a λ lysogen derivative of S17-1 (*pro thi recA hsdR* (r$^-$ m$^+$) Tp$^R$ Sm$^R$ Km$^S$ [Ω RP4-2-Tc::Mu-Km::Tn*7*]) expressing Π protein from *pir* gene of R6K | (Simon *et al.*, 1983) |

[a] Coordinates are according to the published SGI1 sequence AF261825

**Table S2.** Relevant features of plasmids used in this study.

| Plasmid name | Relevant features[a] | Reference |
|---|---|---|
| R55 | IncC Type2, tra+, Cm$^R$, Flo$^R$, Sul$^R$, Ap$^R$, Km$^R$, Gm$^R$ | (Chabbert *et al.*, 1972) |
| R55$^{\Delta Tn6187}$ | R55 derivative, Tn*6187* was deleted, tra+, Cm$^R$, Flo$^R$, Sul$^R$, Ap$^S$, Km$^S$, Gm$^S$ | (Kiss *et al.*, 2019) |
| pBluescript II-SK(+) | pMB1-based Ap$^R$ cloning vector | (Short *et al.*, 1988) |
| pEMBL19 | pMB1-based Ap$^R$ cloning vector | (Dente *et al.*, 1983) |
| pCP20 | Thermo-inducible FLP recombinase expression (λ p$_R$::*FLP*), temperature-sensitive pSC101 replication system, λ *cI857*; Ap$^R$, Cm$^R$ | (Cherepanov and Wackernagel, 1995) |
| pKD3 | R6Kγ-based PCR template plasmid with FRT-flanked *cat* gene for one-step recombination gene-KO Cm$^R$, Ap$^R$ | (Datsenko and Wanner 2000) |
| pKD46 | Ap$^R$ ara-inducible expression vector of λ Red recombinase, temperature-sensitive pSC101 replication system | (Datsenko and Wanner 2000) |
| pSG76-K | R6Kγ-based Km$^R$ replicon | (Pósfai *et al.*, 1997) |
| pAHG36 | A pMSZ949 derivative carrying a single A insertion after the 18080 bp SGI1 position in mob$_{SGI1}$, which causes frameshift mutation (fs4) in S021, S022 and two short ORFs. | this work |
| pFOL1372 | A pJK708 derivative containing the 15444-19843bp (S015-S023) region of SGI1 including the entire mob$_{SGI1}$. | (Kiss *et al.*, 2019) |
| pJKI88 | p15A-based Km$^R$ cloning vector deriving from pACYC177 (Rose, 1988). | (Kiss and Olasz, 1999) |
| pJKI332 | pBluescript II-SK(+) containing a Km$^R$ gene (Ap$^R$Km$^R$). | (Kiss *et al.*, 2019) |
| pJKI391 | p15A-based Km$^R$ expression vector with P$_{tac}$ promoter deriving from pJKI88. | (Kiss *et al.*, 2015) |
| pJKI708 | Sm$^R$ derivative of the p15A-based cloning vector, pJKI88 | (Hegyi *et al.*, 2017) |
| pJKI773 | pFOL1372 derivative carrying a KO mutation in S021, which replaces the 17809-17891 bp segment of mob$_{SGI1}$. | this work |
| pJKI780 | 16447-18680 bp mob$_{SGI1}$ region of SGI1 cloned in pJK708. | (Kiss *et al.*, 2019) |
| pJKI781 | 16447-18140 bp region of SGI1 cloned in pJK708 (the 3'part of mob$_{SGI1}$ including the promoters P3-P6 is deleted from the 3' end of *oriT*). | (Kiss *et al.*, 2019) |
| pJKI783 | The 16447-18680 bp mob$_{SGI1}$ region cloned in pBluescript II-SK(+). | (Kiss *et al.*, 2019) |
| pJKI879 | pET22b+ (Novagen) derivative (Ap$^R$), containing the *mpsAB* (S020-S019) operon under the control of P$_{T7}$. | this work |
| pJKI881 | pJKI391 derivative, containing ORF S021 under the control of P$_{tac}$. | this work |
| pJKI882 | pJKI391 derivative, containing the *mpsAB* (S020-S019) operon under the control of P$_{tac}$. | this work |
| pJKI990 | ColE1-based cloning vector for β-gal assays, containing promoterless *lacZ* gene preceded by pHP45Ω (Prentki and Krisch, 1984) and *rrnB*T1T2 terminators. | (Kiss *et al.*, 2015) |

| | | |
|---|---|---|
| pJKI1125 | A pMNI36 derivative carrying the $P_{cat}$ promoter upstream of the 17775 bp SGI1 position. | this work |
| pJKI1126 | The 17687-18680 bp region of SGI1 cloned in pBluescript II-SK(+) containing a $Km^R$ gene ($Ap^RKm^R$). | this work |
| pJKI1127 | The 17775-18680 bp region of SGI1 cloned in pBluescript II-SK(+) containing a $Km^R$ gene ($Ap^RKm^R$). | this work |
| pMNI11 | A pMSZ949 derivative where the -35 and -10 box of $P_{S022}$ were eliminated by sequence replacement. | this work |
| pMNI18 | A pSG76-K derivative template plasmid for one-step KO mutagenesis containing the *rrnB*T1T2 terminators. | this work |
| pMNI30 | The 17713-18458 bp region of SGI1 cloned in pJKI88. | this work |
| pMNI31 | The 17713-18370 bp region of SGI1 cloned in pJKI88. | this work |
| pMNI32 | The 17713-18412 bp region of SGI1 cloned in pJKI88. | this work |
| pMNI34 | The 17687-18680 bp region of SGI1 cloned in pJKI88. | this work |
| pMNI36 | The 17775-18680 bp region of SGI1 cloned in pJKI88 | this work |
| pMNI37 | The 17713-18511 bp region of SGI1 cloned in pJKI88. | this work |
| pMNI39 | The 17713-18332 bp region of SGI1 cloned in pJKI88. | this work |
| pMNI40 | The 17741-18511 bp region of SGI1 cloned in pJKI88.. | this work |
| pMNI41 | A $Km^R$ derivative of pJKI871 (Kiss *et al.*, 2019), carrying the $oriT_{SGI1}$ (18016-18140 bp region of SGI1). | (Kiss *et al.*, 2019) |
| pMNI42 | The 17713-18140 bp region of SGI1 cloned in pJKI88. | this work |
| pMNI46 | A pMNI30 derivative carrying the $P_{cat}$ promoter upstream of the 18458 bp SGI1 position. | this work |
| pMNI47 | A pMNI39 derivative carrying the $P_{cat}$ promoter upstream of the 18332 bp SGI1 position. | this work |
| pMNI48 | A pMNI42 derivative carrying the $P_{cat}$ promoter upstream of the 18140 bp SGI1 position. | this work |
| pMNI52 | A pMNI31 derivative carrying the $P_{cat}$ promoter upstream of the 18370 bp SGI1 position. | this work |
| pMNI53 | A pMNI32 derivative carrying the $P_{cat}$ promoter upstream of the 18370 bp SGI1 position. | this work |
| pMNI71 | The 17741-18511 bp region of SGI1 cloned in pJKI88. | this work |
| pMSZ946 | pJKI990-derivative β-galactosidase tester plasmid containing the non-coding upstream region of S022 (18330-18681 bp) fused to the promoterless *lacZ* gene. | this work |
| pMSZ949 | The 16447-18680 bp $mob_{SGI1}$ region cloned in pJKI88. | (Kiss *et al.*, 2019) |
| pMSZ957 | pMSZ949 derivative containing a single T insertion at 17816th SGI1 position, which generates a new StuI site and cause frameshift (fs1) in S021 and in a short ORF in the complementary strand. | (Kiss *et al.*, 2019) |
| pMSZ958 | pMSZ949 derivative containing a single C insertion at 17857th SGI1 position, which generates a new PvuI site and cause frameshift (fs2) in S021 in its second, in-frame Met codon and in a short ORF in the complementary strand. | this work |
| pMSZ967 | pMSZ949 derivative containing a TT insertion at 17990th SGI1 position, which generates a new HindIII site and cause frameshift (fs3) in S021 and three small overlapping ORFs and in a short ORF in the complementary strand. | this work |

[a] Coordinates are according to the published SGI1 sequence AF261825

**Table S3.** List of oligonucleotides used in this study.

| Primer | Sequence (5'-3')[a] | Reference |
|---|---|---|
| delS021for | tccgtatccggtggtaagtgaaacgcagtttttttaattagctctgcatgcGTGTAGGCTGGAGCTGCTTC | this work |
| delS021rev | cagtttgttccgtcggaacagaccagccaatggaatgagcagttcgctccCATATGAATATCCTCCTTAGTTC | this work |
| KO_promS022for | tagatttggaaaaatgctgttttgggccagctaccgtttacaGTGACACTATAGAATACAAGCTTGC | this work |
| KO_promS022rev | catcacccaaaagccatcgaaaagccactttctgatgaaCTCAGAAGAACTCGTCAAGAAGG | this work |
| delRNA3for | cgaactgctcattccattggctggtctgttccgacggaacaaactgccagcaGTGTAGGCTGGAGCTGCTTC | this work |
| delRNA4for | cgctgggatcttgcccaaacacaggacagttaaacccgatgtccacggtcatGTGTAGGCTGGAGCTGCTTC | this work |
| delS022rev | aggggcactcctccgtctaaaacctatctccccggaggaaaatcagatgtCATATGAATATCCTCCTTAGTTC | (Kiss *et al.*, 2019) |
| delS022upstream_rev | attgtaaacggtagctggcccaaaacagcatttttccaaatctaaggcgtCATATGAATATCCTCCTTAGTTC | this work |
| deloriTfor | gtgtcattctttggaaaggaaagcgcgaagcgcgctaaccgccgaaggcgGTGTAGGCTGGAGCTGCTTC | this work |
| deloriTrev | aagagggcctccctcatccgtcagaacgagtgctggatttccggcttactCATATGAATATCCTCCTTAGTTC | (Kiss *et al.*, 2019) |
| lacZoutE | gggaattcgacggccagtgaatccgtaat | (Kiss *et al.*, 2019) |
| oriTfor | aactgcagtataattcgcgcacattcgtg | (Kiss *et al.*, 2019) |
| promcatfor | aatctcgagaacttttggcgaaaatgagacg | this work |
| promcatrev | attgtcgacaagcttttagcttccttagctcctgaaaatc | this work |
| pucfor24 | cgccagggttttcccagtcacgac | this work |
| S021for | aactgcagtgatccccctaactctgttaatc | this work |
| S021for2_B | atggatcctgttactttgttggcccgtcc | this work |
| S021for3_B | cgggatccagtgaaacgcagttttttaattagc | this work |
| S022Ndefor | gatgatcatatgtttatcatcattgtgcttgca | this work |
| S022promfor_Nc | aaccatgggattttcctccggggagatag | this work |
| S022promseq | gaatgcacaaacgcaggcagc | this work |
| S022promrev_P | aactgcagtaagttagttgagcatccaaac | (Kiss *et al.*, 2019) |
| S022prommut | cagctaccgtttacaattcat**cgcggccg**caatctgatagtaac**tgacc**gttcatcagaaagtggcttttc | this work |
| S022promrev | aagaattcgtaagttagttgagcatccaaac | (Kiss *et al.*, 2019) |
| S022promrev2 | atgaattctaaacggtagctggccc | this work |
| S022promrev3 | aagaatcccctcatccgtcagaacgag | this work |
| S022promrev4 | ttaagcttgaattcgccactttctgatgaacttgacgttac | this work |
| S022promrev5 | ttaagcttgaattcttgccttcacttcgttaaccctc | this work |
| S022promrev6 | ttaagcttgaattcggcactcctccgtctaaaacctatc | this work |
| SGI1_S021promseq | gaccgtggacatcgggtttaac | this work |
| sgi1_17858for_PvuISph | ctgcatgctaaggccaacgctgggatcttgcccaaacacaggacagttaaacccgat**c**gtccacggtc | this work |
| sgi1_18080fs_PvuI | tagagcccttgaggctcaaggcttccg**a**tcgggggctctac | this work |
| sgi_orf021for | ttggatccatatgctaaggccaacgctgggatcttg | this work |
| sgi_orf021rev | aactgcagggatcctatgctccaccccgtctctg | this work |
| sgi_orf020for | aacatatgcgttcagagcggactaatc | this work |
| sgi_orf019rev | aactgcagggatccttaatcagcagagccggtgtttttttg | this work |
| SGI1orf020_17119for | gaacagtgcgcgcccggccac | (Kiss *et al.*, 2019) |
| SGI1orf021_18003revH | ttagcgcgcttcgc**aa**gctttcctttcc | this work |
| T3_SGI_17741for | *aattaaccctcactaaaggg*tgttactttgttggcccgtcc | this work |
| T7_SGI_17994rev | *taatacgactcactataggg*ttcgcgctttcctttccaaag | this work |
| T3_SGI_18219for | *aattaaccctcactaaaggg*aacgcgacatagctgtcag | this work |
| T7_SGI_18628rev | *taatacgactcactataggg*cctaccactcaagaatcgttcag | this work |

[a] Uppercase shows the template plasmid sequence in primers used for producing the KO amplicons. Restriction cleavage sites are underlined, bold indicates the replaced or inserted bases in the mutagenesis primers, italycs show the promoter sequence of T3/T7 polymerases.

**Supplementary Methods.** Construction of plasmids.

pAHG36: The 18053-18680 bp region of SGI1 was amplified with sgi1_18080fs_PvuI –S022promrev primers. The resulting amplicon was digested with BglI and used to replace the corresponding wt BglI fragment of pMSZ949.

pJKI773: The KO mutation in ORF S021 was generated by the one-step gene inactivation method in pFOL1372. In the resulting plasmid, pJKI770, the 17809-17891 bp region in S021 was replaced with the $Cm^R$ cassette amplified from pKD3 using delS021for-delS021rev primers. The $Cm^R$ gene then was deleted with XbaI digestion and religation of the plasmid backbone leading to pJKI773. In the final sequence the replacement of the 83-bp tract following the $4^{th}$ bp of S021 with the 84-bp pKD3-derived sequence causes frameshift and several stop codons in ORF S021.

pJKI879: The promoterless *mpsAB* (S020-019) operon was cut out with NdeI-BamHI from plasmid pJKI876 (Kiss *et al.*, 2019) and ligated into the NdeI-BamHI site of pET22b+ (Novagen).

pJKI881: ORF S021 was amplified with sgi_orf021for- sgi_orf021rev primers and the PstI-digested amplicon was ligated into the PstI-SmaI site of pBluescript II SK(+). After sequencing, the NdeI-BamHI fragment (contaning S021) of the resulting plasmid pJKI875 was ligated into the corresponding site of pJKI391 (Kiss *et al.*, 2015).

pJKI882: The promoterless *mpsAB* (S020-019) operon was cut out with NdeI-BamHI from pJKI876 (Kiss *et al.*, 2019) and ligated into the corresponding site of pJKI391 (Kiss *et al.*, 2015).

pJKI1125: The 126 bp region containing the promoter of *cat* gene was amplified with promcat_for-promcat_rev primers and the amplicon was digested with XhoI-HindIII and ligated to the corresponding site of pGEM-7Zf(+) (Promega). The XbaI-BamHI fragment of the resulting plasmid pMNI19 was ligated into the XbaI-BamHI site of pMNI10 (see pMNI36) leading to pMNI20. Then, the EcoRI-XbaI fragment of pMNI20 was ligated into the corresponding site of pJKI88.

pJKI1126: The 16447-17687 bp region including *mpsA* and *mpsB* was deleted from the whole $mob_{SGI1}$ in pJKI783 (Kiss *et al.*, 2019) by BspEI-SmaI digestion. The remaining part of the plasmid was religated after filling in the protruding BspEI end with Klenow polymerase resulting in plasmid pMNI1. Then, the $Km^R$ gene cassette from pJKI332 (Kiss *et al.*, 2019) was cut out with BamHI-NotI and ligated to the corresponding site of pMNI1.

pJKI1127: The 17775-18680 bp region of SGI1 was amplified with S021for3_B-S022promrev primers and the amplicon was digested with EcoRI-BamHI and ligated to the corresponding site of pBluescript II KS(+) resulting in plasmid pMNI10. Then, the $Km^R$ gene cassette from pJKI332 (Kiss *et al.*, 2019) was cut out with BamHI-NotI and ligated to the corresponding site of pMNI10.

pMNI11: The 18446-18680 bp region of SGI1was amplified with primers S022promrev-S022prommut and the amplicon was used in a second PCR as megaprimer with the oriTfor primer. The resulting amplicon then was digested with BglI and used to replace the corresponding wt BglI fragment of pMSZ949.

pMNI18: The *rrnB*T1T2 terminator cassette from pJKI988 (Kiss *et al.*, 2015) was transferred into pSG76-K (Pósfai *et al.*, 1997) with BamHI-PstI.

pMNI30: The 17713-18458 bp region of SGI1 was amplified with S022promrev2-S021for primers, the amplicon was digested with EcoRI-PstI and ligated to the EcoRI-PstI site of pBluescript II SK(+) (pMNI5), then the EcoRI-PstI fragment of pMNI5 was ligated into the corresponding site of pJKI88.

pMNI31: The 17713-18370 bp region of SGI1 was amplified with S021for-S022_promrev6 primers and the amplicon was cloned into the HindIII-PstI site of pBluescript II SK(+). The resulting plasmid pMNI26 was then digested with EcoRI-PstI and the fragment was ligated into the corresponding site of pJKI88.

pMNI32: The 17713-18412 bp region of SGI1 was amplified with S021for-S022_promrev5 primers and the amplicon was digested with HindIII-PstI and ligated to the HindIII-PstI site of pBluescript II SK(+). The resulting plasmid pMNI27 was digested with EcoRI-PstI and the fragment was ligated into the corresponding site of pJKI88.

pMNI34: The EcoRI-BamHI fragment of pMNI1 (see pJKI1126) was cloned in the corresponding site of pJKI88.

pMNI37: The 17713-18511 bp region of SGI1 was amplified with S021for-S022promrev4 primers and the amplicon was cloned into the HindIII-PstI site of pBluescript II SK(+) (pMNI25). The EcoRI-PstI fragment of pMNI25 was ligated into the corresponding site of pJKI88.

pMNI36: The EcoRI-PstI fragment of pMNI10 (see pJKI1127) was cloned in the corresponding site of pJKI88.

pMNI39: The 17713-18332 bp region of SGI1 was amplified with S021for-S022Ndefor primers and the amplicon was cloned into the PstI-HincII site of pBluescript II SK(+) (pMNI7). Then, the XhoI-PstI fragment of pMNI7 was cloned in the SalI-PstI site of pJKI88.

pMNI40: The 17741-18511 bp region of SGI1 was amplified with S021for2B-S022promrev4 primers and the amplicon was digested with EcoRI-BamHI and ligated to the corresponding site of pBluescript II SK(+) resulting in pMSZ985, then the same fragment was transferred with EcoRI-BamHI from pMSZ985 into the corresponding site of pJKI88.

The EcoRI-BamHI fragment of containing the 17741-18511 bp region of SGI1 was cloned in the corresponding site of pJKI88.

pMNI42: The 17713-18180 bp region of SGI1 was amplified with S021for-S022promrev3 primers and the amplicon was digested with PstI and cloned into the PstI-HincII site of pBluescript II SK(+). Then the XhoI-PstI fragment of the resulting plasmid pMNI6 was cloned in the SalI-PstI site of pJKI88.

pMNI46: The 126-bp $P_{cat}$ promoter region amplified with promcat_for-promcat_rev primers was digested with XhoI-HindIII and ligated into the corresponding site of pMNI5 (see pMNI30). Then, the 17713-18458 bp SGI1 region completed with $P_{cat}$ was cut out from the resulting plasmid pMNI45 with XhoI-PstI and ligated into the SalI-PstI site of pJKI88.

pMNI47: The 126-bp $P_{cat}$ promoter region amplified with promcat_for-promcat_rev primers was digested with XhoI-SalI and ligated into the XhoI site of pMNI7 (see pMNI39). Then, the 17713-18332 bp SGI1 region complemented with $P_{cat}$ was cut out from the resulting plasmid pMNI43 with XhoI-PstI and ligated into the SalI-PstI site of pJKI88.

pMNI48: The 126-bp $P_{cat}$ promoter region was amplified with promcat_for-promcat_rev primers. The amplicon was digested with XhoI-SalI and ligated into the XhoI site of pMNI6 (see pMNI42) in the corresponding orientation. Then, the 17713-18140 bp SGI1 region completed with $P_{cat}$ was cut out from the resulting plasmid pMNI21 with XhoI-PstI and ligated into the SalI-PstI site of pJKI88.

pMNI52: The XhoI-HindIII fragment ($P_{cat}$) from pMNI19 (see pJKI1125) was ligated into the corresponding site of pMNI27 (see pMNI32). Then, the 17713-18370 bp SGI1 region completed with $P_{cat}$ was cut out from the resulting plasmid pMNI26_cat with XhoI-PstI and ligated into the SalI-PstI site of pJKI88.

pMNI53: The XhoI-HindIII fragment ($P_{cat}$) from pMNI19 (see pJKI1125) was ligated into the corresponding site of pMNI26 (see pMNI31). Then, the 17713-18412 bp SGI1 region completed with $P_{cat}$ was cut out from the resulting plasmid pMNI27_cat with XhoI-PstI and ligated into the SalI-PstI site of pJKI88.

pMNI71: The17741-18680 bp region of SGI1 was amplified with S021for2_B-S022promrev primers and the amplicon was cloned into the EcoRI-BamHI site of pBluescript II KS(+). The resulting plasmid pMNI9 was digested with EcoRI-BamHI and the fragment was ligated into the corresponding site of pJKI88.

pMSZ946: The 18327-18681 bp region of SGI1 was amplified with primers S022promfor_Nc and S022promrev_P. The amplicon was digested with NcoI-PstI and ligated to the corresponding site of pJKI990.

pMSZ958: The 17801-18681 bp region of SGI1 was amplified with primers S021for_PvuI,Sph and S022promrev_P. Then, the SphI-PstI fragment of pMSZ949 was replaced by the SphI-PstI-digested amplicon.

pMSZ967: The 17118-18003 bp region of SGI1 was amplified with primers SGI1orf020_17119for and SGI1orf021_18003revH. The the BssHII fragment of pMSZ949 was replaced by the BssHII fragment of the amplicon.

**Supplementary References**


Chabbert, Y.A., Scavizzi, M.R., Witchitz, J.L., Gerbaud, G.R., and Bouanchaud, D.H. (1972) Incompatibility Groups and the Classification of f- Resistance Factors. *J Bacteriol* **112**: 666–675.

Cherepanov, P.P., and Wackernagel, W. (1995) Gene disruption in Escherichia coli: TcR and KmR cassettes with the option of Flp-catalyzed excision of the antibiotic-resistance determinant. *Gene* **158**: 9–14.

Datsenko, K.A., and Wanner, B.L. (2000) One-step inactivation of chromosomal genes in Escherichia coli K-12 using PCR products. *Proc Natl Acad Sci U S A* **97**: 6640–5 http://www.pubmedcentral.nih.gov/articlerender.fcgi?artid=18686&tool=pmcentrez&rendertype=abstract. Accessed July 11, 2014.

Dente, L., Cesareni, G., and Cortese, R. (1983) pEMBL: A new family of single stranded plasmids. *Nucleic Acids Res* **11**: 1645–1655.

Gibson, T.J. (1984) Studies on the Epstein-Barr virus genome. Thesis. .

Gonzy-Treboul, G., Karmazyn-Campelli, C., and Stragier, P. (1992) Developmental regulation of transcription of the Bacillus subtilis ftsAZ operon. *J Mol Biol* **224**: 967–979.

Hegyi, A., Szabó, M., Olasz, F., and Kiss, J. (2017) Identification of oriT and a recombination hot spot in the IncA/C plasmid backbone. *Sci Rep* **7**: 10595 http://www.nature.com/articles/s41598-017-11097-0.

Kiss, J., Nagy, B., and Olasz, F. (2012) Stability, entrapment and variant formation of Salmonella genomic island 1. *PLoS One* **7**: e32497 http://www.pubmedcentral.nih.gov/articlerender.fcgi?artid=3285670&tool=pmcentrez&rendertype=abstract.

Kiss, J., and Olasz, F. (1999) Formation and transposition of the covalently closed IS 30 circle : the relation between tandem dimers and monomeric circles. *Mol Microbiol* **34**: 37–52.

Kiss, J., Papp, P.P., Szabó, M., Farkas, T., Murányi, G., Szakállas, E., and Olasz, F. (2015) The master regulator of IncA/C plasmids is recognized by the Salmonella Genomic island SGI1 as a signal for excision and conjugal transfer. *Nucleic*

*Acids Res* **43**: 8735–8745.

Kiss, J., Szabó, M., Hegyi, A., Douard, G., Praud, K., Nagy, I., *et al.* (2019) Identification and Characterization of oriT and Two Mobilization Genes Required for Conjugative Transfer of Salmonella Genomic Island 1. *Front Microbiol* **10**: 1–16 https://www.frontiersin.org/article/10.3389/fmicb.2019.00457/full.

Pósfai, G., Koob, M.D., Kirkpatrick, H.A., and Blattner, F.R. (1997) Versatile insertion plasmids for targeted genome manipulations in bacteria: Isolation, deletion, and rescue of the pathogenicity island LEE of the Escherichia coli O157:H7 genome. *J Bacteriol* **179**: 4426–4428.

Prentki, P., and Krisch, H.M. (1984) In vitro insertional mutagenesis with a selectable DNA fragment. *Gene* **29**: 303–313.

Rose, R.E. (1988) The nucleotide sequence of pACYC184. *Nucleic Acids Res* **16**: 355 http://www.ncbi.nlm.nih.gov/pmc/articles/PMC334639/.

Sambrook, J., Fritsch, E.F., and Maniatis, T. (1989) *Molecular Cloning: A Laboratory Manual. Cold Spring Harbor Laboratory Press, Cold Spring Harbor, NY.* .

Short, J.M., Fernandez, J.M., Sorge, J.A., and Huse, W.D. (1988) Lambda ZAP: A bacteriophage lambda expression vector with in vivo excision properties. *Nucleic Acids Res* **16**: 7583–7600.

Simon, R., Priefer, U., and Pühler, A. (1983) A Broad Host Range Mobilization System for In Vivo Genetic Engineering: Transposon Mutagenesis in Gram Negative Bacteria. *Bio/Technology* **1**: 784–791 http://www.nature.com/doifinder/10.1038/nbt1183-784.

Szabó, M., Murányi, G., and Kiss, J. (2021) IncC helper dependent plasmid-like replication of Salmonella Genomic Island 1. *Nucleic Acids Res* **49**: 832–846.
