# Supplementary material for: Salmonella Genomic Island 1 requires a self‐encoded small RNA for mobilization
Source: Mol Microbiol. 2021 Nov 25;116(6):1533–51. doi: 10.1111/mmi.14846 (PMC9299015; doi:10.1111/mmi.14846)
Supplement: Supplementary file 2 — Fig S8 [file MMI-116-1533-s001.pdf]

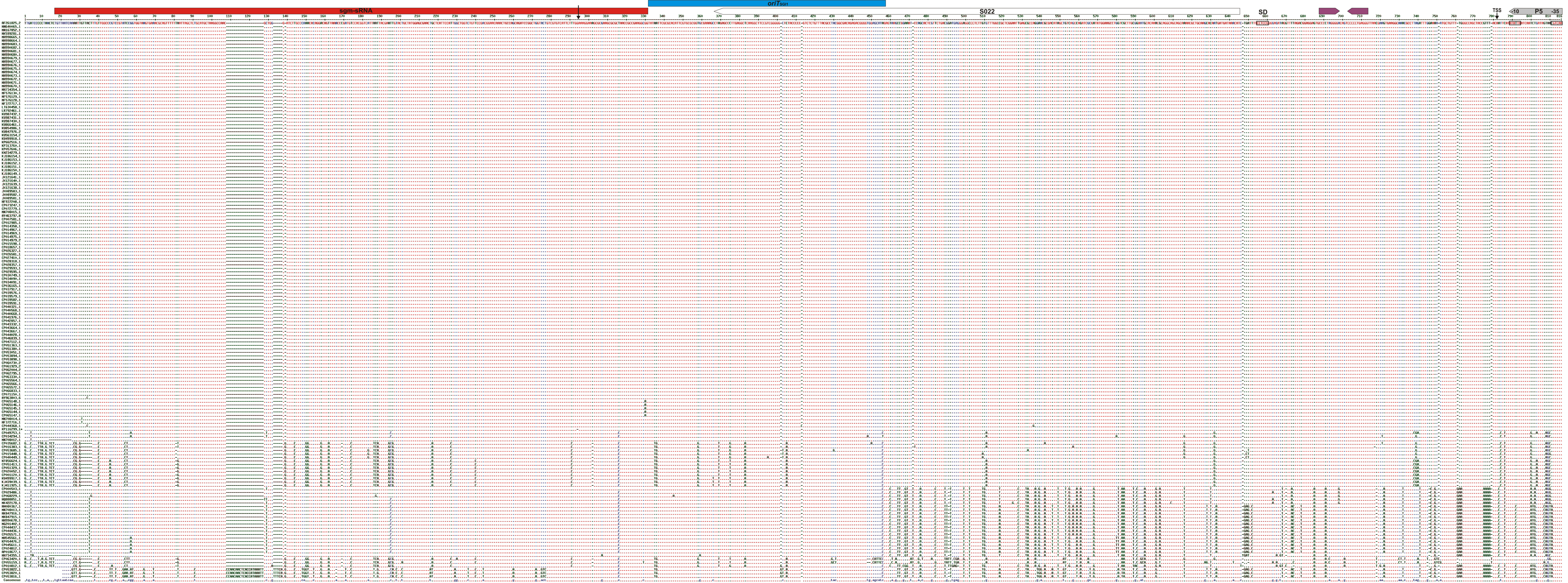

Figure S8. Alignment of the 3' end of mob_{SGI1} region encoding the primary transcript of sgm-sRNA. Homologous sequences to the 1266-2048 bp segment of mob_{SGI1} from 159 SGI1-family elements are aligned. The SGI1-related islands were retrieved from GenBank using the SGI1 backbone as query sequence (Kiss *et al.*, 2019). Promoter P5, TSS, the terminator-like IR, the SD-box and the coding sequence of ORF S022, *oriT*$_{SGI1}$ and the maximum range of the core domain of sgm-sRNA are indicated above the alignment. * The insertion site of a large IS26-based transposon in SGI1-B2 from *Proteus mirabilis* strain PmSC17 (KP116299.1) is indicated by a vertical arrow.
